# Activation of Toll-like receptor 7/8 encoded by the X chromosome alters sperm motility and provides a novel simple technology for sexing sperm

**DOI:** 10.1371/journal.pbio.3000398

**Published:** 2019-08-13

**Authors:** Takashi Umehara, Natsumi Tsujita, Masayuki Shimada

**Affiliations:** Graduate School of Biosphere Science, Hiroshima University, Higashi-Hiroshima, Japan; University of Michigan, UNITED STATES

## Abstract

In most mammals, the male to female sex ratio of offspring is about 50% because half of the sperm contain either the Y chromosome or X chromosome. In mice, the Y chromosome encodes fewer than 700 genes, whereas the X chromosome encodes over 3,000 genes. Although overall gene expression is lower in sperm than in somatic cells, transcription is activated selectively in round spermatids. By regulating the expression of specific genes, we hypothesized that the X chromosome might exert functional differences in sperm that are usually masked during fertilization. In this study, we found that Toll-like receptors 7/8 (TLR7/8) coding the X chromosome were expressed by approximately 50% of the round spermatids in testis and in approximately 50% of the epididymal sperm. Especially, TLR7 was localized to the tail, and TLR8 was localized to the midpiece. Ligand activation of TLR7/8 selectively suppressed the mobility of the X chromosome–bearing sperm (X-sperm) but not the Y-sperm without altering sperm viability or acrosome formation. The difference in sperm motility allowed for the separation of Y-sperm from X-sperm. Following in vitro fertilization using the ligand-selected high-mobility sperm, 90% of the embryos were XY male. Likewise, 83% of the pups obtained following embryo transfer were XY males. Conversely, the TLR7/8-activated, slow mobility sperm produced embryos and pups that were 81% XX females. Therefore, the functional differences between Y-sperm and X-sperm motility were revealed and related to different gene expression patterns, specifically TLR7/8 on X-sperm.

## Abstract 2

The Toll-like receptors TLR7 and TLR8 are encoded by the X chromosome and expressed in X-containing sperm but not Y-containing sperm. TLR7/8 ligands suppress the motility of X-containing sperm, indicating that this receptor can differentially affect sperm function on the basis of the sex chromosome they bear.

## Introduction

Male germ cells proliferate by mitosis, develop as primary spermatocytes, and eventually, by the process of meiosis, become mature sperm. In the first meiotic division, the primary spermatocyte divides into two secondary spermatocytes, which then cleave leading to the generation of four haploid round spermatids that contain either the X or Y chromosome [1, 2]. The subsequent differentiation of round spermatids to sperm is called as spermiogenesis [3], and during this process, the round spermatid undergoes dynamic morphological changes, including the nuclear condensation, acrosome formation, and the elongation of the sperm flagella [3,4]. As indicated by studies using knockout mouse models, the morphological changes associated with spermiogenesis are associated with the expression of various genes, including *Tekt*, *Tnp*, and *Gba2* in the round spermatids [5–7]. Thus, active gene transcription occurs on chromosomes, including sex chromosomes, in haploid male germ cells, and some of them are essential for cell survival [8]. Using the cytoplasmic bridges between spermatids, cytoplasm including RNAs and proteins are shared to rescue Y chromosome bearing sperm (Y-sperm) [8,9]. It has been reported that the bridge works in spermatid at early stage of spermiogenesis, and the high levels of RNA polymerase II are detected in this stage [10,11]. However, RNA polymerase II is still detected in later stages of spermiogenesis [11], indicating that the unique features of sperm can be distinguished not only by the presence of the X or Y chromosome but also by expression of distinct genes encoded by each sex chromosome.

However, mouse Y chromosome encodes fewer than 700 genes, whereas mouse X chromosome encodes over 3,000 genes [12, 13]. The X chromosome contains some unique genes, such as *Taz* encoding tafazzin that is a transcriptional regulator activated by physical stimulation [14], *Xiap* encoding X-linked inhibitor of apoptosis protein that suppresses the activation of apoptosis signaling [15], and *G6pdx* encoding X-linked glucose-6-phosphate 1-dehydrogenase that catalyzes the oxidative pentose–phosphate pathway [16]. Thus, it is assumed that the X chromosome, as well as autosomes, play an essential role in regulating the somatic and germ cell functions. However, both X chromosome bearing sperm (X-sperm) and Y-sperm are produced and are equally functional, with no apparent difference between X-sperm and Y-sperm during fertilization in vivo or in conventional in vitro fertilization (IVF) procedures.

Despite these functional similarities, differences in X- and Y-sperm mobility have been observed. Specifically, although Sarkar and colleagues (1984) reported that the mobility of human sperm in the stationary fluid was not different between X-sperm and Y-sperm, the movement of X-sperm, but not Y-sperm, shifted to the nearly straight path in a flow-stream protocol [17]. Additionally, the motility of Y-sperm rapidly decreased compared with that of X-sperm in specific in vitro conditions, such as low pH, high temperature, and high oxidative stress [18], whereas the motility of X-sperm decreased in the high-pH incubation conditions [18,19]. These observations indicated that, the motility patterns of X-sperm and Y-sperm differ under specific in vitro (and presumably in vivo) conditions and might be related to the transcription of specific genes that impact the unique motility patterns of X-sperm or Y-sperm. However, these analyses have not yet been done.

Our previous studies showed that sperm express specific Toll-like receptors capable of responding to specific ligands [20,21]. Thus, one might predict that if sperm expressed specific environmental sensors (i.e., receptors) encoded by either the X chromosome or Y chromosome and if these sensors were activated selectively by specific conditions, then functional differences in motility might be manifested between X-sperm and Y-sperm. In this study, we sought to identify specific genes encoded by each sex chromosome in sperm that might account for the potential functional differences in between X-sperm and Y-sperm. Based on evidence obtained from this approach, we developed a novel fast and simple technology for determining Y-sperm versus X-sperm that, without specific and elaborate sorting systems, could be used to selectively separate Y-sperm or X-sperm and generate male and female pups in mice.

## Results

### TLR7/8 are encoded by genes on the X chromosome in mouse sperm

From RNA sequence data of mouse sperm (Accession Number: DRA007935), 492 genes were selected as expressed genes from X chromosome in mouse sperm. Eighteen of the X chromosome genes encode receptors. Six of these receptors (*Tlr8*, *Ar*, *Gpr174*, *Tlr7*, *Gpr34*, and *Edr2a*) have specific ligands; the other 12 receptors have common ligands that also bind to receptors encoded by autosomes. The presence of these receptor mRNAs was confirmed by reverse transcription polymerase chain reaction (RT-PCR) (S1 Fig). Additionally, the marker genes of somatic cells, *Hoxa1* and *Hoxb1*, were not detected in RNAs extracted from mouse sperm by RT-PCR (S1 Fig).

**Fig 1:**
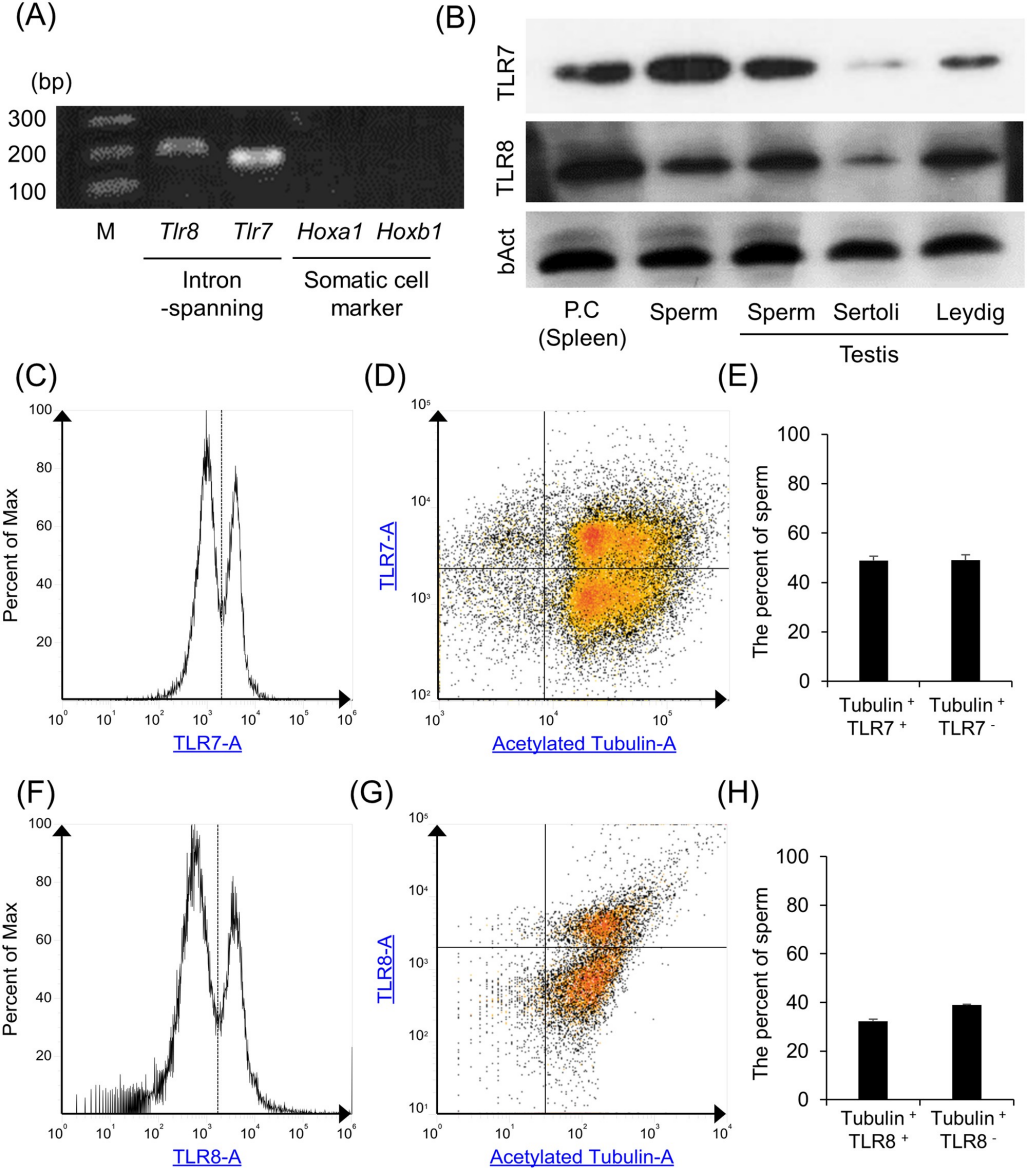
The expression of TLR7/8 encoded by the X chromosome in mouse sperm. (A) Presence of *Tlr7*/*8* mRNAs in mouse sperm. mRNA was extracted from sperm and used for RT-PCR analyses using intron-spanning primer sets. To check the contamination of RNA extracted from epididymis in the sperm RNA sample, *Hoxa1* and *Hoxb1* were used as the marker of somatic cells. cDNA products were resolved on 2% (w/v) agarose gels. (B) Expression of TLR7/8 in mouse sperm. Sperm lysates were prepared for western analyses using an anti-TLR7 or anti-TLR8 antibody. β− Actin was used as a loading control. Lysates prepared from spleen tissue were used as PCs. Results are representative of three biological experiments. (C,D) TLR7 histogram (C) and the density plot between TLR7 and acetylated tubulin (D) of mouse sperm. Sperm were incubated with the anti-TLR7 antibody conjugated with Alexa Fluor 647 and anti-acetylated tubulin antibody, which is a marker of sperm tail, and then were used for FCM. Dotted line and solid line indicated the borderline between positive cells and negative cells. (E) Percent of TLR7-positive and acetylated tubulin–positive sperm (TLR7^+^/Tubulin^+^) and TLR7-negative and acetylated tubulin–positive sperm (TLR7^−^/Tubulin^+^). The experiment was repeated with three biological replicates. Values represent the mean ± SEM of three replicates. Data associated with this figure can be found in the supplemental data file (S1 Data). (F,G) TLR8 histogram (F) and the density plot between TLR8 and acetylated tubulin (G) of mouse sperm. Sperm were incubated with the anti-TLR8 antibody and anti-acetylated tubulin antibody, which is a marker of sperm tail, and then were used for FCM. Dotted line and solid line indicated the borderline between positive cells and negative cells. (H) Percent of TLR8-positive and acetylated tubulin–positive sperm (TLR8^+^/Tubulin^+^) and TLR8-negative and acetylated tubulin–positive sperm (TLR8^−^/Tubulin^+^). The experiment was repeated three biological replicates. Values represent the mean ± SEM of three replicates. Data associated with this figure can be found in the supplemental data file (S1 Data). FCM, flow cytometry; TLR7/8, Toll-like receptor 7/Toll-like receptor 8.

In our previous study, we have reported that TLR2 and TLR4 are detected in human, boar, and mouse sperm, and the TLR2 and TLR4 ligands suppress survivability, motility, and capacitation status of mammalian sperm [20,21]. However, the function of TLR7/8 coding in X chromosome on sperm function remained unclear. Thus, in this study, we focused on TLR7/8 as candidates to understand the potential differences of X-spermatids and Y-spermatids and to develop new sexing technology. The presence of *Tlr*7*/8* mRNAs were detected by RT-PCR using intron-spanning primer; on the other hand, the presence of *Hoxa1* and *Hoxb1* mRNAs were not detected (Fig 1A). The expressions of TLR7/8 protein were analyzed by western analyses, flow cytometry (FCM), and immunofluorescence (IF). Both TLR7/8 proteins were expressed in spleen and were used as a positive control (PC). TLR7/8 were also present in epididymal sperm, testicular sperm cells, and Leydig cells but were weak in Sertoli cells (Fig 1B). By FCM, the rates of TLR7- or TLR8-positive sperm were also analyzed using anti-acetylated tubulin antibody, a marker of the sperm flagella. The distinct two peaks of TLR7 or TLR8 were observed, and the peak under 1,100 was defined as negative sperm, and the other was defined as positive sperm (Fig 1C and 1F). Almost all of the cells were stained by anti-acetylated tubulin antibody, and few of them were negative that were contaminated blood cells. The percent of the double-stained cells (TLR7^+^/Tubulin^+^ or TLR8^+^/Tubulin^+^) was nearly equivalent to the percent of the sperm stained only by anti-acetylated tubulin antibody (TLR7^−^/Tubulin^+^ or TLR8^−^/Tubulin^+^) (TLR7^+^/Tubulin^+^ versus TLR7^−^/Tubulin^+^: 48.8% ± 1.8% versus 49.0 ± 2.1% [Fig 1D and 1E] and TLR8^+^/Tubulin^+^ versus TLR8^−^/Tubulin^+^: 32.3% ± 0.8% versus 38.9% ± 0.3% [Fig 1G and 1H]).

**Fig 2:**
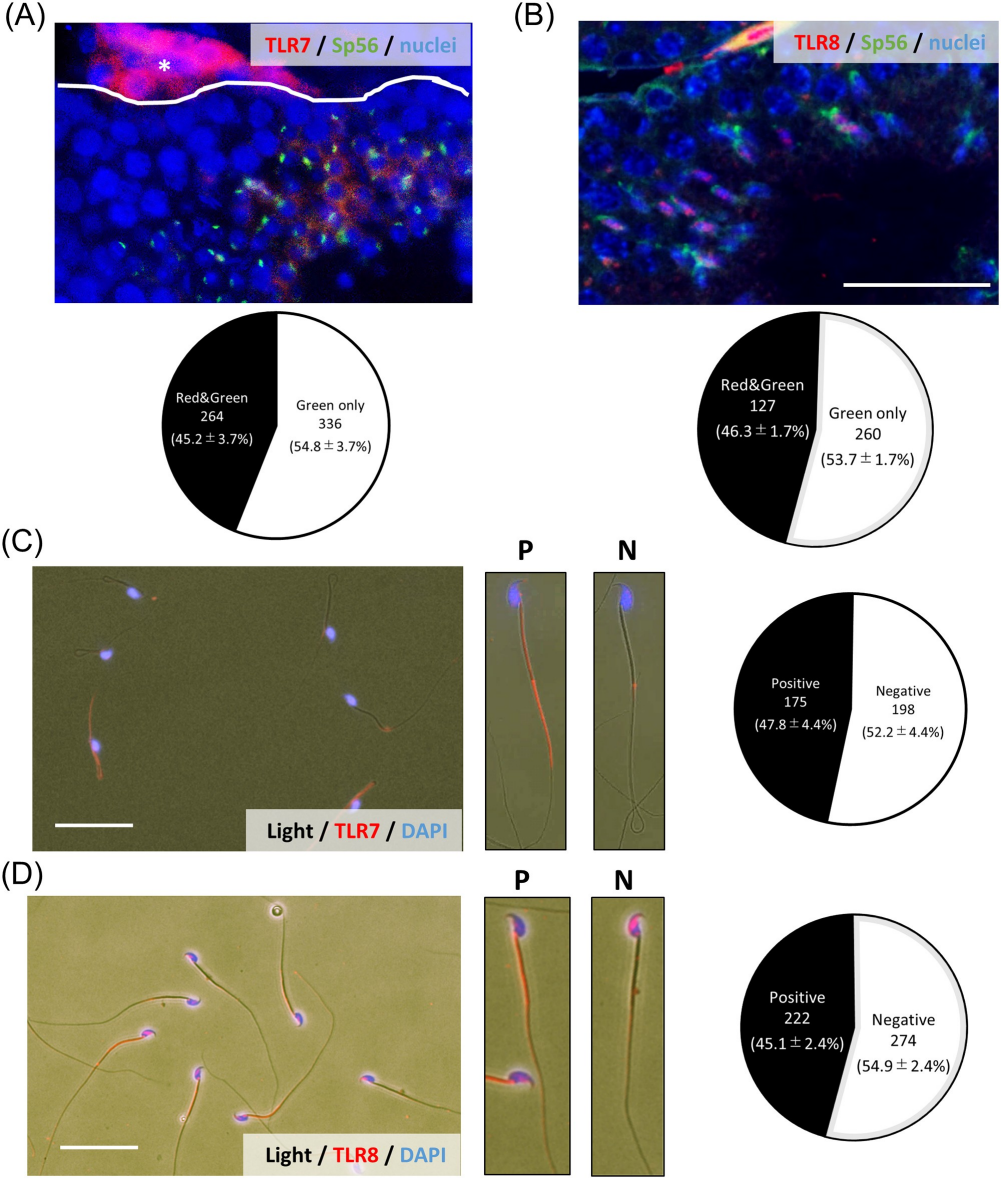
The localization of TLR7/8 in mouse testis and sperm. (A) Localization of TLR7 in mouse testis. Cross-sections of mouse testes were stained with antibodies to visualize either TLR7 (red) or Sp56 (green), a marker of acrosome in round spermatid and sperm at 12 weeks. White lines delineate the border of the seminiferous tubule. Lower chart showed that the percent of Sp56 single positive cells or TLR7-Sp56 double positive cells in the seminiferous tubule. The number of positive cells in a section per testis was counted, and a total of three different samples were analyzed. Data associated with this figure can be found in the supplemental data file (S1 Data). *Leydig cell. Scale bar indicates 100 μm. (B) Localization of TLR8 in mouse testis. Cross-sections of mouse testes were stained with antibodies to visualize either TLR8 (red) or Sp56 (green), a marker of acrosome in round spermatid and sperm at 12 weeks. Lower chart showed that the percent of Sp56 single positive cells or TLR8-Sp56 double positive cells in the seminiferous tubule. The number of positive cells in a section per testis was counted, and a total of three different samples were analyzed. Data associated with this figure can be found in the supplemental data file (S1 Data). Scale bar indicates 100 μm. (C) Localization of TLR7 in mouse sperm collected from the epididymis. Sperm were collected from the epididymis into HTF medium and then air dried. Smears were incubated with the anti-TLR7 antibody and then a secondary antibody. P: the high magnification image of the sperm with a P signal of TLR7. N: the high magnification image of sperm with N for TLR7. Right figure showed that the percent of TLR7 positive/negative sperm. Scale bar indicates 10 μm. The number of positive sperm was counted, and a total of four different samples were analyzed. Data associated with this figure can be found in the supplemental data file (S1 Data). (D) Localization of TLR8 in mouse sperm collected from the epididymis. Sperm were collected from the epididymis into HTF medium and then air dried. Smears were incubated with the anti-TLR8 antibody and then a secondary antibody. P: the high magnification image of the sperm with a P signal of TLR8. N: the high magnification image of sperm with N for TLR8. Right figure showed that the percent of TLR8 positive/negative sperm. Scale bar indicates 10 μm. The number of positive sperm was counted, and a total of three different samples were analyzed. Data associated with this figure can be found in the supplemental data file (S1 Data). HTF, human tubal fluid; N, negative; P, positive; Sp56, formally known as a common name of zona pellucida 3 receptor (ZP3R); TLR7/8, Toll-like receptor 7/Toll-like receptor 8.

By IF, TLR7-positive signals were observed in about half of the round spermatids that were double stained with zona pellucida 3 receptor (ZP3R; formerly called Sp56), a marker of round spermatids during spermiogenesis (Fig 2A). The percent of double-positive cells was 45.2% ± 3.7% of Sp56-positive cells (Fig 2A). TLR8-positive signals were also detected in round spermatids (Fig 2B). The percent of double-positive cells was 46.3% ± 1.7% of Sp56-positive cells (Fig 2B). With IF, half of round spermatids collected from seminiferous tubule were stained by anti-TLR7 antibody by FCM (S2 Fig). Additionally, by Immuno-FISH using the probe of X chromosome (X-paint) and anti-TLR7 antibody, the positive signals of TLR7 were only observed in X chromosome–bearing cells (S3 Fig); however, some of X chromosome–bearing cells were TLR7 negative because the cells collected from seminiferous tubule were not only spermatid but also spermatogonia cells.

In mouse sperm collected from cauda epididymis, TLR7-positive signals were detected in the flagella of sperm; on the other hand, TLR8-positive signals were detected in the midpiece of sperm. However, the percent of TLR7- and TLR8-positive sperm were each about 50% (TLR7: 47.8% ± 4.4%, TLR8: 45.1% ± 2.4%; Fig 2C and 2D). (Negative control was showed in S4 Fig).

### TLR7/8 ligands suppressed progressive motility of the sperm bearing the X chromosome

**Fig 3:**
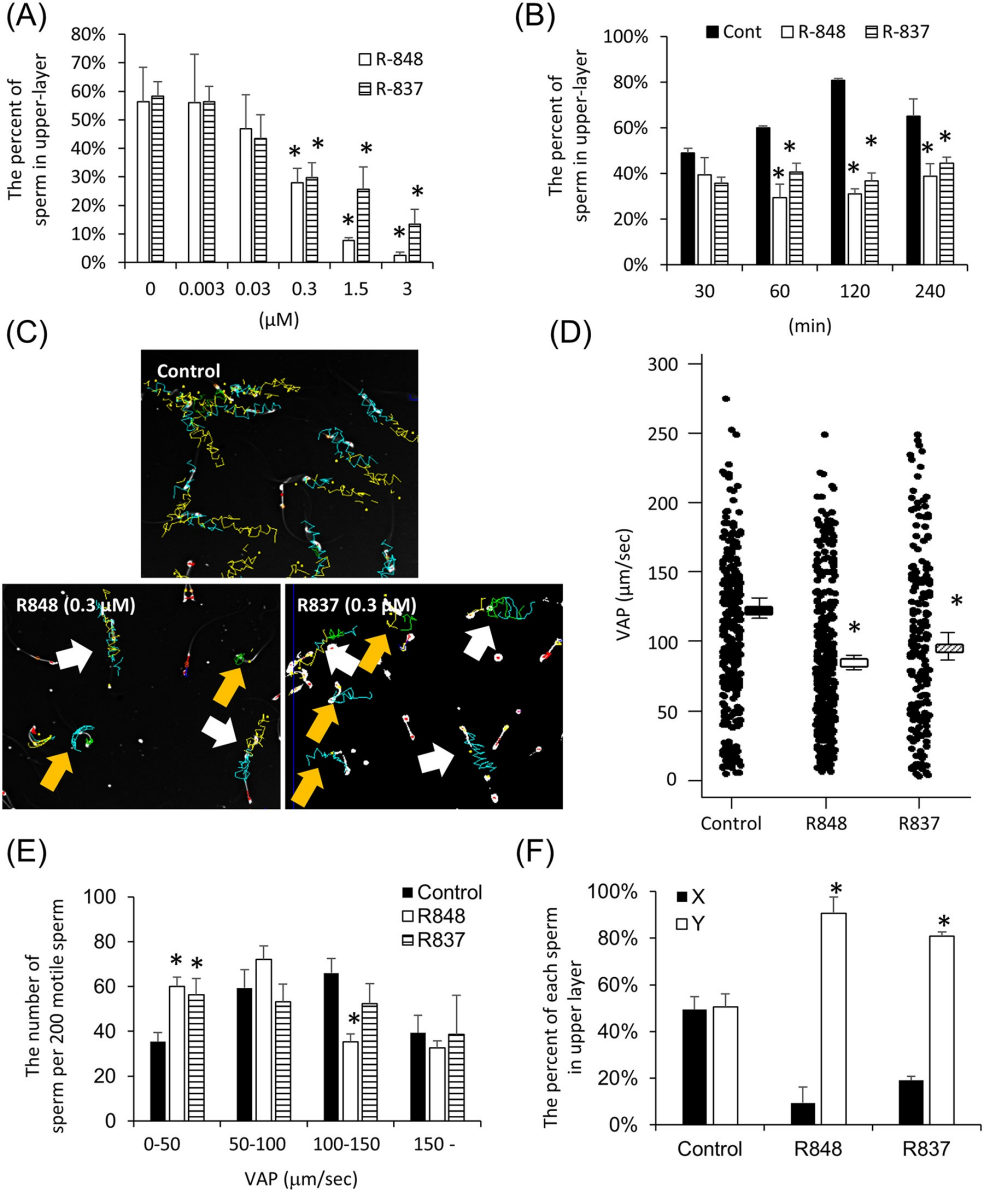
TLR7/8 ligands suppressed progressive motility the sperm bearing the X chromosome. (A) Percentages of swim-up sperm when sperm were cultured in HTF medium with R848, a TLR7/8 ligand, or R837, a TLR7-specific ligand. Sperm were collected from the mouse epididymis and cultured in HTF medium with several doses of R848 or R837 for 1 hr. The numbers of sperm in the upper layer and the number of total sperm were counted at 1 hr. The percent of swim-up sperm was then calculated. The experiment was repeated four biological replicates. Values represent the mean ± SEM of four replicates. Data associated with this figure can be found in the supplemental data file (S1 Data). (B) Percentages of swim-up sperm when sperm were cultured in HTF medium with 0.3 μM R848 or R837. Sperm were collected from the mouse epididymis and cultured in HTF medium with/without 0.3 μM R848 or R837 for a maximum of 240 min. The numbers of sperm in the upper layer and total sperm were counted at each time point. The percent of swim-up sperm was then calculated. The experiment was repeated three biological replicates. Values represent the mean ± SEM of three replicates. Data associated with this figure can be found in the supplemental data file (S1 Data). (C) Tracks of sperm incubated with/without 0.3 μM R848 or 0.3 μM R837 for 1 hr, determined using the CASA system. White arrows indicated the progressive sperm (VAP > 70 μm/sec). Yellow arrows indicated the slow sperm (VAP < 30 μm/sec). (D, E) Variability of average-path velocity (VAP) of sperm after R848 or R837 treatment (D) and the number of the sperm exhibiting each VAP, such as 0–50, 50–100, 100–150, and over 150 μm/s€(E). Sperm were incubated with 0.3 μM R848 or R837 and then analyzed by CASA technology. The experiment was repeated three biological replicates (more than 200 individual trajectories in each treatment group were analyzed per mouse). Values represent the mean ± SEM. *Denotes significant differences compared with the control. Data associated with this figure can be found in the supplemental data file (S1 Data). (F) Effect of R848 or R837 on sex selection of mouse sperm. Sperm were incubated with 0.3 μM R848 or R837 for 60 min, and then upper layer was collected. After centrifuging, the pellet was used for DNA extraction, and real-time PCR using the specific primer recognized X chromosome or Y chromosome. The experiment was repeated three biological replicates. Values represent the mean ± SEM. *Denotes significant differences compared with the control. Data associated with this figure can be found in the supplemental data file (S1 Data). CASA, computer-assisted sperm analysis; HTF, human tubal fluid; R837, Imiquimod, selective agonist for TLR7; R848, Resiquimod; TLR7/8, Toll-like receptor 7/Toll-like receptor 8; VAP, average-path velocity.

Because TLR7 and TLR8 encoded on the X chromosome were expressed in sperm and both have common ligands, sperm were incubated with Resiquimod (R848) that binds both TLR7 and TLR8 or Imiquimod (R837), a specific ligand of TLR7. Using a motility assay (S5 Fig), the percent of highly active sperm that swim to an upper layer (swim-up sperm) was significantly decreased by the addition of either ligand in a dose-dependent manner from 0.3 μM to 3 μM (Fig 3A). The reduced mobility was also evident when sperm were incubated with 0.3 μM for more than 60 min (Fig 3B). As a result of computer-assisted sperm analysis (CASA), the tracks of some sperm incubated with 0.3 μM R848 or R837 were shorter than those of sperm incubated without TLR7/8 ligand (control) (Fig 3C). The average-path velocity (VAP) of sperm was also significantly decreased by R848 or R837. Although the number of sperm in the groups with approximately 50–100 μm/sec or over 150 μm/sec VAP was not altered by the treatment with R848 (Fig 3D and 3E), the number of sperm significantly increased when VAP was observed under 50 μm/sec, and significantly decreased VAP was observed from 100 μm/sec to 150 μm/sec (Fig 3E). Additionally, by the treatment of R837, the number of sperm in the groups under 50 μm/sec also significantly decreased, indicating that TLR7/8 suppressed the velocity of some but not all sperm. The percent of X-sperm in the swim-up layer (in upper 200 μl of 500 μl) was dramatically decreased, whereas the percent of Y-sperm in upper layer was dramatically increased by the treatment of either R848 or R837 over 60 min (Fig 3F). Importantly, treatment with R848 did not affect either sperm viability or the acrosome status of sperm (S6 Fig), indicating that sperm after the treatment with R848 kept the fertilization ability. Additionally, the slow motility of sperm mediated by R848 was reversed by the removing of R848 in the culture media (S7 Fig).

### TLR7/8 ligands suppress ATP production by glycolysis via a GSK3α/β–hexokinase pathway

**Fig 4:**
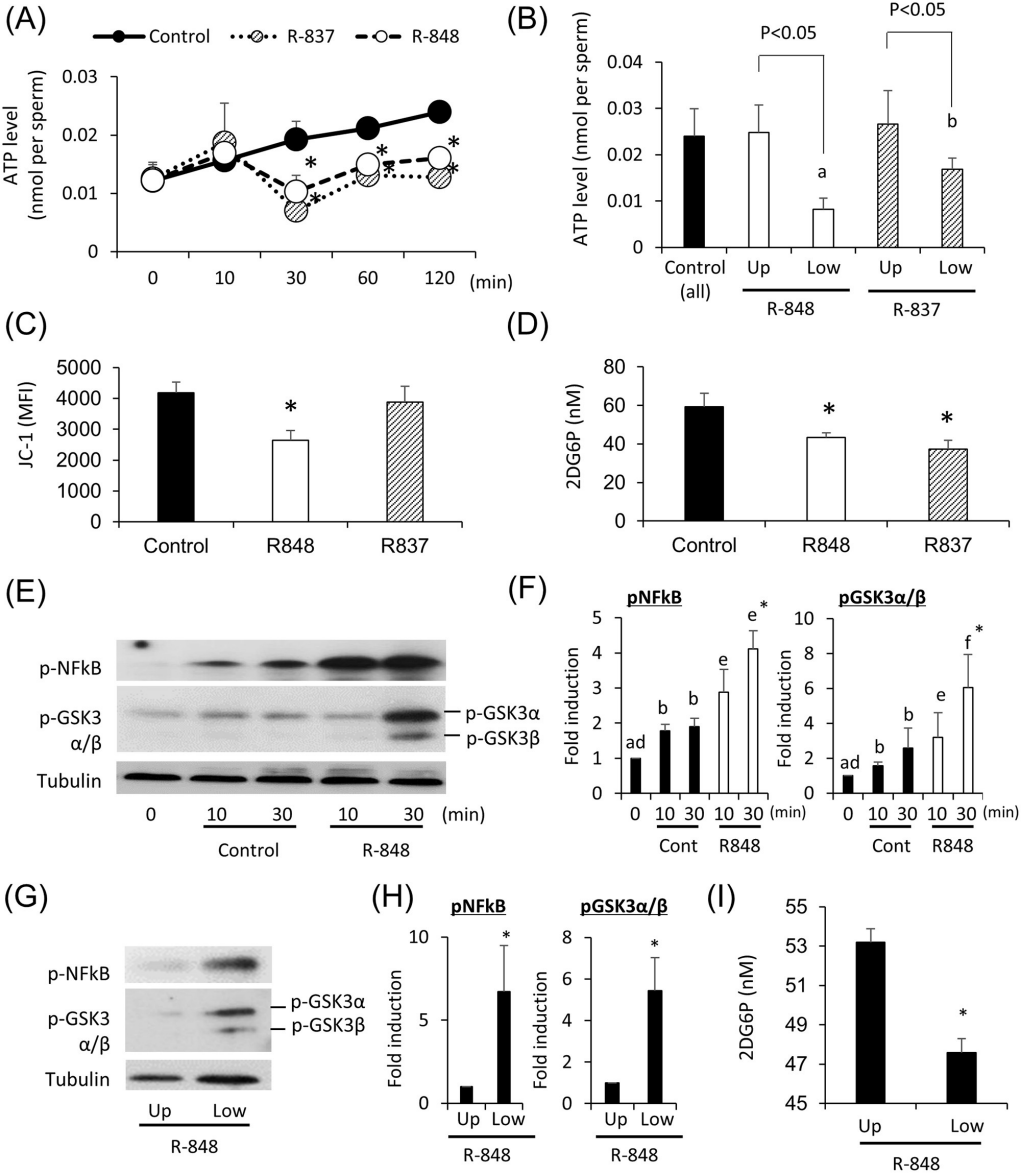
The treatment with TLR7/8 ligands suppressed ATP production in glycolysis via GSK3 α/β-hexokinase pathway. (A) Changes in ATP levels of sperm after culture in HTF medium with/without 0.3 μM R848 or R837. Sperm were collected from the mouse epididymis and then treated with 0 (control), 0.3 μM R848, or 0.3 μM R837 for maximum 120 min. The experiment was repeated three biological replicates. Values represent the mean ± SEM of three replicates. *P < 0.05 compared with the level of the control. Data associated with this figure can be found in the supplemental data file (S1 Data). (B) Comparison of ATP levels between the sperm in upper layer and the sperm in lower layer after R848 treatment. Sperm were collected from the mouse epididymis and then treated with 0 (control: all sperm) or 0.3 μM R848 or R837 for 60 min. After incubation, the medium containing sperm was separated into the upper layer and lower layer. The sperm were collected and ATP levels analyzed. The experiment was repeated three biological replicates. Values represent the mean ± SEM of three replicates. ^a,b^; significant differences were observed between R848 treatment groups and R837 group. Data associated with this figure can be found in the supplemental data file (S1 Data). (C) Change of mitochondrial activity after R848 or R837 treatment using JC-1 kit. Sperm were collected from the mouse epididymis and then treated with 0 (control) or 0.3 μM R848 or R837 for 60 min. After incubation, sperm were incubated with 500 mL 1x working solution at 37 °C for 30 min in dark, the mitochondrial activity was analyzed by flow cytometry using a filter with a bandwidth of 574/26 nm and measured as the MFI of JC-1 orange aggregates. A total of 50,000 sperm events were analyzed. The experiment was repeated three biological replicates. Values represent the mean ± SEM of four replicates. *P < 0.05 compared with the level of the control. Data associated with this figure can be found in the supplemental data file (S1 Data). (D) Effect of R848 or R837 on the activity of hexokinase. Sperm collected from the epididymis were incubated with/without 0.3 μM R848 for 30 min in HTF medium containing 2-deoxy-glucose. Because 2-deoxy-glucose was converted to 2DG6P, that is not used for glycolysis, the activity of hexokinase was measured by the accumulation of 2DG6P in the sperm. *P < 0.05 compared with the control. Data associated with this figure can be found in the supplemental data file (S1 Data). (E) Induction of the phosphorylation of NFκB and GSK3α/β in mouse sperm by R848. Sperm collected from the epididymis were incubated with 0.3 μM R848 for 0, 10, or 30 min. Phosphorylation was detected by an anti-phospho NFκB antibody or anti-phospho GSK3a/b antibody. Tubulin was used as a loading control. Results are representative of three independent experiments. (F) Intensity of the phosphorylation of NFκB and GSK3α/β induced by R848. The intensity of all bands was analyzed using a Gel-Pro Analyzer (Media Cybernetics, MD, USA). Values are the mean ± SEM of three replicates. ^a,b^; the phosphorylation levels were significantly increased by incubation in control group as compared with those in sperm before incubation (0 min). ^d,e, d-f^; the phosphorylation levels were significantly increased by the incubation with R848 as compared with those in sperm before incubation (0 min). *P < 0.05 compared with the control at same time point. Data associated with this figure can be found in the supplemental data file (S1 Data). (G) Phosphorylation of NFκB and GSK3α/β occurred in the sperm present in lower-layer after R848 treatment. Sperm collected from the epididymis were incubated with 0.3 μM R848 for 30 min. After incubation, the medium containing sperm was separated into upper layer and lower layer. Cell lysates were prepared for western analyses using an anti-phospho NFκB antibody or anti-phospho GSK3α/β antibody. Tubulin was used as a loading control. Results are representative of three independent experiments. (H) Intensity of the phosphorylation of NFκB and GSK3α/β induced in lower-layer by R848 treatment. The intensity of all bands was analyzed using a Gel-Pro Analyzer (Media Cybernetics, MD, USA). Values are the mean ± SEM of three replicates. *P < 0.05 compared with the control at same time point. Data associated with this figure can be found in the supplemental data file (S1 Data). (I) Effect of R848 on the activity of hexokinase in lower sperm. Sperm collected from the epididymis were incubated with/without 0.3 μM R848 for 30 min in HTF medium containing 2-deoxy-glucose. Because 2-deoxy-glucose was converted to 2DG6P, that is not used for glycolysis, the activity of hexokinase was measured by the accumulation of 2DG6P in the sperm. *P < 0.05 compared with the control. Data associated with this figure can be found in the supplemental data file (S1 Data). 2DG6P, 2-deoxy-glucose-6-phosphate; GSK3α/β, glycogen synthase kinase 3α/β; HTF, human tubal fluid; MFI, mean fluorescence intensity; TLR7/8, Toll-like receptor 7/Toll-like receptor 8.

**Fig 5:**
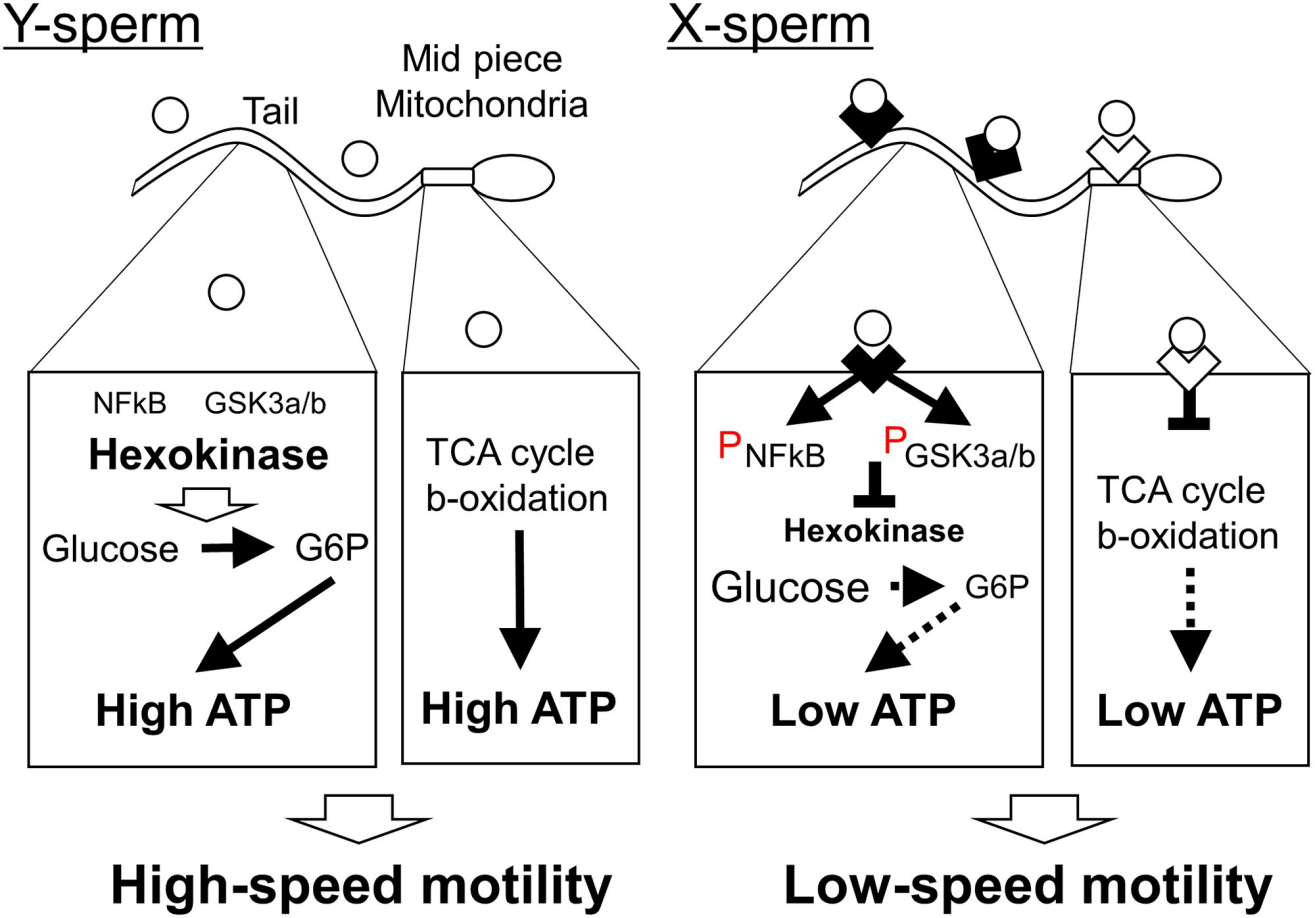
The model of ATP production in Y-sperm and X-sperm under TLR7/8-ligand condition. In Y-sperm, mitochondrial and glycolytic ATP production occur regardless of the presence of TLR7/8 ligand (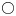). Thus, Y-sperm showed high-speed motility. On the other hand, in X-sperm, the activation of TLR8 localized in the midpiece (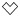) suppressed mitochondrial ATP production. Additionally, the activation of TLR7 localized in the sperm tail (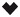) phosphorylated NFκB and GSK3α/β and then suppressed the activity of hexokinase. As a result, ATP production in X-sperm was decreased under TLR7/8-ligand condition. Thus, only X-sperm showed low-speed motility under TLR7/8-ligand condition. GSK3α/β, glycogen synthase kinase 3α/β; TLR7/8, Toll-like receptor 7/Toll-like receptor 8; NFκB, nuclear factor-kappa B; X-sperm, X chromosome–bearing sperm; Y-sperm, Y chromosome–bearing sperm.

Because sperm velocity is regulated by intracellular ATP levels, we examined the effect of TLR7/8 ligands on the production of ATP in sperm. The results showed that ATP levels in sperm were significantly decreased by incubation with both TLR7/8 ligands for 30 min or greater (Fig 4A). The change was primarily observed in slow mobility sperm present in the lower layer after swim-up test with TLR7/8 ligand, and ATP level after R848 treatment was significantly lower than that after R837 treatment (Fig 4B). Because the production of ATP is regulated by the mitochondrial tricarboxylic acid (TCA) cycle, β-oxidation, and/or cytoplasmic glycolysis, the mitochondrial activity and glycolysis activity were measured. JC-1, which is a marker of mitochondrial activity, was significantly decreased by the treatment of R848 but not R837 (Fig 4C). To determine the activity of hexokinase, a rate-limiting enzyme of glycolysis, we measured the levels of 2-deoxy-6-phosphate-glucose and observed that the levels were significantly suppressed by both TLR7/8 ligand (Fig 4D), indicating that the suppression of mitochondrial activity was a specific phenotype of the activation of TLR8, and the suppression of glycolysis activity was a common phenotype in both TLR7 and TLR8. As a consequence, phosphorylation of NFκB, a downstream target of TLRs signaling and the phosphorylation of GSK3α/β, an inhibitor of glycolysis was increased by the ligand R848 (Fig 4E and 4F). These changes were observed in slow-mobility sperm present in the lower layer after the swim-up test with R848 (Fig 4G, 4H and 4I). Therefore, the activation of TLR8 localized in the midpiece of sperm suppressed mitochondrial activity, and the activation of TLR7 localized in the tail of sperm suppressed cytoplasmic glycolysis (Fig 5).

### TLR7/8 ligand activation of TLR7/8 enabled sex preselection for IVF

**Fig 6:**
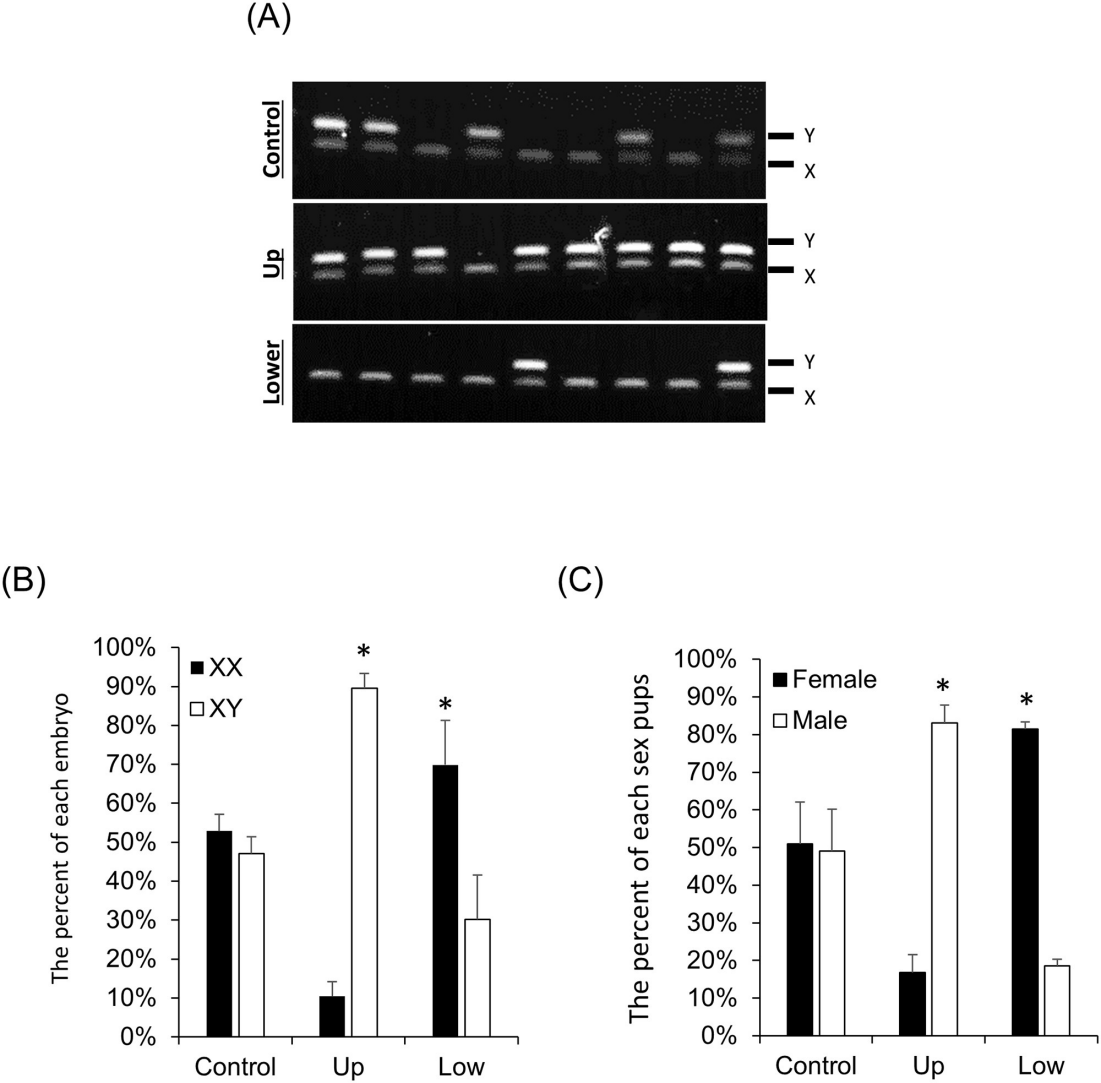
The treatment with TLR7/8 ligand enabled sex preselection for IVF. (A) The sexing of blastocyst embryos derived from IVF using the sperm treated with R848. Upper image: sex ratios of embryos using the sperm incubated in HTF medium. Middle image: sex ratios of embryos using the upper-layer sperm incubated in HTF medium contained 0.3 μM R848. Lower image: sex ratios of embryos using the lower-layer sperm incubated in HTF medium contained 0.3 μM R848. Upper band (187 bp) denotes the presence of the Y chromosome, and lower band (143 bp) denotes the presence of the X chromosome. (B) Ratio of XX embryos or XY embryos in IVF using the sperm treated with R848. Values are the mean ± SEM of four replicates. *P < 0.05 compared with the control. Data associated with this figure can be found in the supplemental data file (S1 Data). (C) Ratio of male or female pups in IVF embryo transfer using the sperm treated with R848. Thirty blastocysts at 3.5 days after insemination were surgically transferred into the uterine horns of 2.5-day-old pseudo-pregnant females. The number of pups was then recorded at birth. Values are the mean ± SEM of at least five replicates. Data associated with this figure can be found in the supplemental data file (S1 Data). HTF, human tubal fluid; IVF, in vitro fertilization; R848, Resiquimod; TLR7/8, Toll-like receptor 7/Toll-like receptor 8.

The method for separating between X-sperm and Y-sperm is shown in S5 Fig. The X-sperm or Y-sperm separated by R848 were collected, the content of X- and Y-sperm was determined, and the sperm was used for in vitro fertilization (IVF). The fertilization rate of mature oocytes and the percentage of blastocysts stage embryos per pro-nuclei (PN) embryos were not altered between control sperm, upper-layer sperm treated with R848 (Up), or lower-layer sperm treated with R848 (Low) (S8 Fig). Seventy-seven blastocyst embryos were obtained by IVF using Up sperm; 68 of these were XY embryos and nine were XX embryos (XY versus XX; 89.6% ± 3.7% versus 10.3% ± 3.7%) (S1 Table, Fig 6A and 6B). Conversely, 83 blastocyst embryos were obtained by IVF using Low sperm; 58 of these were XX embryos; 25 were XY embryos (XX versus XY; 69.8% ± 11.4% versus 30.1% ± 11.4%) (S1 Table, Fig 6A and 6B). The number of pups delivered from blastocysts generated using Up sperm and Low sperm did not differ significantly from that of controls (S8 Fig). When embryos were generated by Up sperm and used for embryo transfer, 83.1% ± 4.6% of total pups were XY male. When embryos were generated by Low sperm and used for embryo transfer, 81.4% ± 1.9% of total pups were XX female (Fig 6C).

## Discussion

Changes in the expression of specific genes have been documented and shown to occur at distinct stages of spermatogenesis. Comprehensive analyses, such as microarray and RNA sequence analyses, of specific sperm cell types from spermatogonia to round spermatids catalogued the molecular profiles associated with spermatogenesis [22,23], documented the decline in transcriptional activity from round spermatids to sperm [11], and determined that noncoding small RNAs, such as micro RNA and piwi-interacting RNA, in sperm were associated preferentially (exclusively) with the early embryogenesis following fertilization but not before [24]. However, the transcriptome profiles specific to the X and Y chromosomes that might confer inherent and different characteristics of X-sperm and Y-sperm, and perhaps explain sex bias, have not been thoroughly investigated. Therefore, in the present study, we focused on the qualitative, not quantitative, detection of RNAs encoded by sex chromosomes in sperm. Our RNA sequencing data identified 492 genes that were encoded by the X chromosome and only 15 genes by Y chromosome of mouse sperm. Among these genes are members of different classes of receptors that have the potential to mediate sperm responses to factors in their external environment, such as chemokines [25,26], creatine [27], progesterone [28], and pathogens. Thus, functional differences of X-sperm and Y-sperm can be identified and provide a novel approach for specifically selecting X-sperm or Y-sperm.

Members of the TLR family recognize specific pathogens, are expressed in mammalian sperm, and impair fertilization by altering sperm capacitation and hyperactivated motility when semen or uteri are infected with bacteria and/or viruses [20,21]. Therefore, we focused on the receptor-coding genes encoded by the X chromosome that act as sensors that might confer functional differences between X-sperm and Y-sperm and provide a novel approach for specifically selecting X- or Y-sperm. Six receptor genes (*Ar*, *Tlr8*, *Gpr174*, *Tlr7*, *Gpr34*, and *Eda2r*) of the 492 X chromosome–encoded genes bind specific ligands but do not bind to ligands that activate receptors encoded by autosomes.

The functions of the six candidate genes have been reported; mice with a sperm cell–specific *Ar* knockout or mice with genomic disruption of ectodysplasin A-A2 (EDA-A2), a ligand of *Eda2r*, are fertile [29,30]. In an in vitro culture study, lysophosphatidic acid (LPA), a ligand of *Gpr34* and *Gpr174*, induces the acrosome reaction of bull sperm [31]. TLR7/8 are expressed in mouse sperm [32], and the activation of TLR7/8 suppresses the motility of mouse and human sperm [33]. Although these studies indicated the possibility that receptors encoded by the X chromosome receptors might mediate functional differences between X-sperm and Y-sperm, functional differences in X-sperm and Y-sperm that might be related to X chromosome–specific transcription have not previously been determined. Our results, therefore, show for the first time that TLR7/8 are expressed in half of the round spermatids and sperm and that treatment of sperm with TLR7/8–specific ligands dramatically reduced the velocity of X-sperm without affecting that of Y-sperm. These novel observations indicated that TLR7/8 encoded by the X chromosome mediated functional differences between X-sperm and Y-sperm in response to specific activation TLR7/8.

However, it is thought that the cytoplasm in round spermatids is shared between X chromosome–bearing spermatids (X-spermatids) and Y chromosome–bearing spermatids (Y-spermatids) [8,9]. Additionally, the sharing of cytoplasm between X-spermatids and Y-spermatids is required for the survival of Y-spermatids because several survival factors are encoded in only the X chromosome [8–10]. These RNAs/proteins are transferred through the cytosolic bridges between round spermatids. On the other hand, in this study, TLR7/8 were localized in only X-spermatids (S2 and S3 Figs), and all of TLR7/8 positive cells were stained by anti-sp56 antibody, a marker of acrosome, suggesting that these receptors were expressed in spermatids at the latter stage of spermiogenesis. Although we did not observe the proteins forming the cytosolic bridges between round spermatids, such as TEX14, it is one possibility that X chromosome–encoding proteins including TLR7/8 that are expressed at the latter stage of spermiogenesis would not be shared between X-spermatids and Y-spermatids.

The mechanisms by which TLR7/8 activation mediates changes in sperm activity appear to be directly regulated by the production of ATP [34]. Specifically, ligand activated TLR7/8 caused comparatively shorter tracks of sperm movement than those of control sperm in which TLR7/8 were not activated, and this was associated with decreased ATP content in the sperm and the phosphorylation of glycogen synthase kinase 3α/β(GSK3α/β) and nuclear factor-kappa B (NFκB) as known the downstream pathways of TLR7/8 [35]. Although there is no report about the relationship between TLR7/8 and the production of ATP in sperm, the phosphorylation of GSK3α/β decreases the hexokinase enzyme activity and decreases the ATP level in dendritic cells [36,37]. Hexokinase is an limited enzyme of the glycolysis that converts glucose to glucose-6-phosphate [38]. The enzyme that is expressed and localized in the sperm tail [39] is potentially suppressed by TLR7/8 activation in the dendritic cell [36]. On the other hand, it has been also reported that treatment with the GSK3 inhibitor induces acrosome reaction, and the phosphorylation of GSK3 activates the enzyme activity of phosphoprotein phosphatase family that impacts on sperm motility. Therefore, we provide the first evidence that activation of TLR7/8 leads to reduce the levels ATP production via the suppression of hexokinase activity; however, further studies are required for the mechanisms how TLR7/8 regulate hexokinase activity in sperm.

Interestingly, the treatment with R848 that activates both TLR7 and TLR8 suppressed not only hexokinase activity but also mitochondrial activity in sperm. R837 that is selective activator of TLR7 only decreased glycolytic pathway. TLR7 was detected in sperm tail but TLR8 expressed in the midpiece of sperm, which localized in mitochondria, indicating that the activation of TLR8 directly suppressed mitochondrial activity, and TLR7 inhibited hexokinase activity (Fig 5). In our previous study, ATP production in mitochondria regulated the progressive motility in sperm, and the system was independent on glycolysis [40]. Because sperm motility and ATP level were much decreased by the treatment with R848 as compared with those by R837, it is estimated that both TLR7 and TLR8 would regulate ATP generation, which impacts on sperm motility.

The physiological relevance of activating TLR7/8 receptors in sperm has not been thoroughly explored. However, TLR7/8 can be activated by single-strand RNA released from RNA viruses [41], such as Hepatitis C, HIV, and Zika, that are reported to infect the female reproductive tracts, especially the uterus and oviduct [42–44]. RNA-mediated activation of TLR7/8 in reproductive tracts might increase the ratio of XY embryos versus XX embryos. However, the relationship between RNA viral infections and the sex ratio of newborns has not been established. Interestingly, Drew and colleagues (1978) reported that the number of male newborns from couples infected with Hepatitis B Virus (HBV) was higher than those born from the normal couples [45]. Although HBV is a DNA virus and does not directly bind to TLR7/8, the synthesized ligand of TLR7 increases the response against HBV in T-cell and natural killer cells [46]. Immunization with HBV and TLR7/8 agonists induce antigen-specific immune responses in HBV-transgenic mice [47]. Although the molecular mechanisms by which HBV and TLR7/8 interact have remained unclear, the relationship between TLR7/8 and HBV might be one of the reasons for the male sex ratio bias in newborns of the couples infected with HBV. Therefore, an epidemiological study of the relationship between the sex ratio bias and RNA viral infection would determine if there is such an in vivo function of TLR7/8 in sperm.

Based on these observations, we utilized ligand activation of TLR7/8 to provide a convenient, effective, fast, and simple method for the selectively separating Y-sperm from X-sperm by the reducing the velocity of X-sperm. Specifically, the incubation of sperm for 1 hr with TLR7/8 ligands efficiently separated X-sperm from Y-sperm without decreasing fertilization, at least in vitro. Gledhill and colleagues (1983) focused on the length of the sex chromosome and developed the separation method of X-sperm and Y-sperm by cell sorting of sperm stained with Hoechst 33342 [48]. However, this method based on flow cytometry and cell sorting has several limitations. The ultraviolet rays used to detect fluorescence in sperm by flow cytometry decreases sperm fertilization capacity [49]. Furthermore, flow cytometry with a cell-sorting system is too expensive to introduce locally at each farm. Because the expression of the *Tlr7/*8 gene is highly conserved on the mammalian X chromosome [50], our novel protocol using TLR7/8 ligand activation has high promise of being adapted to mammalian reproductive technologies for separating the X-sperm and Y-sperm within a short time and without costly, injurious and time-consuming cell-sorting systems.

In conclusion, TLR7/8 encoded by the X chromosome is expressed in, and localized to, the mid piece and the tail of X-sperm but not the Y-sperm. The addition of TLR7/8 ligands dramatically suppressed the hyperactivated motility of X-sperm without the decreasing of sperm fertilization ability. Thereby, incubating sperm with TLR7/8 ligands permits easy separation of X-sperm and Y-sperm with XY selection attained at more than 90 percent in IVF. Thus, TLR7/8 is specific receptors that differentially impacts the functions of X-sperm but not Y-sperm.

## Materials and methods

### Ethics statement

Animals were housed in the Experiment Animal Center at Hiroshima University under a 14-hr light, 10-hr dark schedule and provided with food and water ad libitum. Animals were treated in accordance with the NIH Guide for the Care and Use of Laboratory Animals, as approved by the Animal Care and Use Committee at Hiroshima University (C18-17, C17-33-2).

### Materials

Pregnant mare serum gonadotropin (PMSG) and human chorionic gonadotropin (hCG) were purchased from Asuka Seiyaku (Tokyo, Japan). Avian myeloblastosis virus (AMV) reverse transcriptase from Promega (Madison, WI, USA), routine chemicals, and reagents were obtained from Sigma-Aldrich (St. Louis, MO, USA) or Nakarai Chemical Co (Osaka, Japan). The TLR7/8 ligands (R848 and R837 were obtained from Novus Biologicals [Littleton, CO, USA] and Enzo Life Sciences [Plymouth Meeting, PA, USA], respectively).

### Animals

Immature female (3 weeks old), adult female (8 to 12 weeks old), and 3-month-old male C57BL/6 mice were obtained from Charles River Laboratories Japan. On day 23 of age, immature female mice were injected intraperitoneally (IP) with 4 IU of eCG to stimulate follicular growth followed by 48 hrs later with 5 IU hCG to stimulate ovulation.

### The treatment of mouse sperm with TLR7/8 ligands and swim test procedure

Sperm were collected from the cauda epididymis into 500 μL of human tubal fluid (HTF) medium. Swim-up test was done according to Umehara and colleagues [27]. The sperm were incubated in HTF medium containing several concentrations of R848 or R837 for different time intervals and then the number of sperm present in either the upper layer or lower layer, and the total number of sperm were counted to calculate the percentage of swim-up or swim-down sperm (S5 Fig). Additionally, the sperm were used for further analysis.

### Analyses of sperm motility using a CASA system

Sperm were incubated in HTF medium containing 0, 0.3 μM R848 or 0.3 μM R837 at 37 °C under 5% CO_2_ in air for 4 hrs. Sperm samples (10 μL) were loaded into 10-μm-deep makler counting chambers. Sperm tracks (0.5 sec, 45 frames) were captured at 60 Hz using a CASA system (HT CASA -Ceros II, Hamiltan Thorne, MA, USA). More than 200 individual trajectories were recorded, as described in our previous study [27]. The progressive sperm were those in which the VAP was higher than 70 μm/sec, and the slow sperm were those in which the VAP was lower than 30 μm/sec.

### IVF in mouse model

IVF was done as described previously [27]. Cumulus-oocyte complexes (COCs) were collected from the oviduct of female mice at 16 hrs after an ovulatory hCG injection. Ten ovulated COCs collected from oviducts were placed in 50 μL of HTF medium for each IVF treatment.

Sperm were collected from the cauda epididymis to 1,000 μL of HTF medium or 0.3 μM TLR7/8 ligand–containing HTF medium. After 60 min of incubation to induce sperm capacitation and to react with TLR7/8 ligand, the upper layer (500 μL) was transferred to a new tube, and then was centrifuged at 37 °C for 5 min. The pellet of upper layer was suspended to HTF medium and was centrifuged at 37 °C for 5 min to remove the TLR7/8 ligand. On the other hands, the lower layer (300 μL) was also transferred to a new tube, and washed similar to the upper layer. After centrifuging, the pellet of sperm was suspended and transferred to fertilization medium at final number of 1,000 sperm per COC (S5 Fig). At 6 hrs after insemination, oocytes were examined for numbers of pro-nuclei to assess the fertilization and were further cultured in developing medium (KSOM+AA, Millipore, Billerica, MA, USA) to analyze blastocyst development. Some blastocysts were collected and were used to sexing of blastocysts using PCR.

### Embryo transfer of mouse

Embryo transfer was done as described by our previous reports [27]. Briefly, 8- to 12-week-old females were mated to vasectomized males and determined to be pseudo-pregnant by the presence of a vaginal plug. Three and a half days after IVF (insemination), 30 blastocysts were surgically transferred into the uterine horn of 2.5-day-old pseudo-pregnant females. The number of pups delivered was recorded.

### Sex determination of blastocyst embryos by PCR

Each of the blastocysts was transferred individually into the tube containing 5 μL of distilled water. Following five cycles of repeated freezing and thawing, the DNA containing supernatant was collected by centrifugation. PCR analyses were performed with KOD FX Neo (TOYOBO Life Science, Osaka, Japan) according to the manufacturer’s instructions. Specific primer sets recognizing either the X chromosome or Y chromosome were generated and used in PCR reactions. The primer sequences were shown in S2 Table. PCR products were resolved on 2% (w/v) agarose gels.

### RNA extraction and RT-PCR

Sperm were collected from cauda epididymis in HTF medium, and were incubated for 30 min. After that the only sperm of upper layer were transferred a new tube to remove the other cells, such as epididymis cells. After centrifuging, total RNA was obtained from mouse sperm using the RNAeasy Mini Kit (Qiagen Sciences, Germantown, MD, USA) according to the manufacturer’s instructions. The total RNAs were also purified as positive controls for RT-PCR from epididymis, spleen, brain, and testis. Total RNA was reverse transcribed using 500 ng poly-dT and 0.25 U avian myeloblastosis virus-reverse transcriptase at 42 °C for 75 minutes and 95 °C for 5 min. RT-PCR analyses were performed with KOD FX Neo (TOYOBO) according to the manufacturer’s instructions. Specific primer pairs used in the RT-PCR reactions are shown in S3 Table; cDNA products were resolved on 2% (w/v) agarose gels.

### Determination of sperm sex ratio by real-time PCR

Total DNA was obtained from sperm using the DNeasy Blood & Tissue Kit (Qiagen Sciences) according to the manufacturer’s instructions. To produce a standard curve, either X or Y amplification product was fractionated by electrophoresis through a 2% agarose gel and then was purified by QIAquick Gel Extraction Kit (Qiagen). DNA and primers that are shown in S2 Table, were added to 15 μL total reaction volume of the Power SYBR Green PCR Master Mix (Applied Biosystems, Foster City, CA). PCR reactions were then performed using the StepOne real time PCR system (Applied Biosystems). Conditions were set to the following parameters: 10 minutes at 95 °C followed by 50 cycles each of 15 sec at 95 °C and 1 minute at 64 °C.

The percentages of X and Y chromosome were calculated by the modified method according to Parati et al. [51]. Briefly, by real-time PCR using several concentrations of X- or Y-amplification products, two lines (the standard curves of X or Y chromosome) were obtained. Additionally, we checked that Ct_X and Ct_Y were near using DNA derived from XY sample, and Ct_X and Ct_Y were far using DNA derived from XX sample. After that, real-time PCR using semen samples was done, and obtained Ct_X and Ct_Y every semen samples. The proportions of sperm bearing each chromosome were calculated by the interpolation of Ct_X, and Ct_Y obtained for sperm sample. The relative amount (*%X* or *%Y*) of X and Y chromosome was calculated using under equitation;

$$\%X=\frac{Amount\;X}{Amount\;X+Amount\;Y},\%Y=\frac{Amount\;Y}{Amount\;X+Amount\;Y},\%X+\%Y=100\%$$

### Western blot analyses

All sample were lysed with RIPA buffer [20 mM Tris (pH 7.5), 150 mM NaCl, 1% (v/v) Nonidet P-40, 0.5% (w/v) sodium deoxycholate, 1 mM EDTA, and 0.1% (w/v) sodium dodecyl sulfate] containing complete protease inhibitors (Roche, Indianapolis, IN). Western analyses were performed according to our previous study [52]. Briefly, extracts (10 μg protein) were mixed with equal volume of 2X SDS sample buffer, resolved by SDS polyacrylamide gel (12.5%) electrophoresis and transferred to PVDF membranes (GE Bioscience, Newark, NJ, USA). Membranes were blocked in Tris-buffered saline and Tween 20 (TBST; 10mM Tris [pH 7.5], 150 mM NaCl and 0.05% [v/v] Tween 20) containing 5% (w/v) nonfat Carnation instant milk (Nestle Co., Solon, OH, USA). Blots were incubated with primary antibodies (Anti-TLR7 antibody [#bs-6601; Bioss, Woburn, MA, USA], anti-TLR8 antibody [#ab180610; Abcam, Cambridge, MA, USA], anti-phospho NFkB antibody [#3031; Cell Signaling, Boston, MA], anti-total NFκB antibody [#3034; Cell Signaling], anti-phospho GSK3α/β antibody [#9331; Cell Signaling], and anti-α/β-tubulin antibody [#2148; Cell Signaling] was used at 1:1,000) overnight at 4 °C. After washing in TBST, Enhanced chemiluminescence (ECL) detection was performed using the ECL system according to the manufacturer’s specifications (GE Bioscience, Newark, NJ, USA) and proper exposure of the blots to Fuji X-ray film (Fujifilm, Tokyo, Japan). The intensity of the bands was analyzed using a Gel-Pro Analyzer (Media Cybernetics, Rockville, MD, USA).

### IF of sperm

Sperm were collected from the cauda of the epididymis with a 27-G syringe and mounted on glass slides and air-dried. The sperm were fixed with 100% methanol for 10 min at room temperature and then permeabilized by 0.1% (v/v) TritonX-100/PBS. Sperm were probed with the primary antibody (anti-TLR7 or anti-TLR8 antibodies were used at 1:100). After washing by 0.3% (v/v) Triton X-100 in PBS (−), the antigens were visualized with Cy3-conjugated goat anti-rabbit IgG (1:200, Sigma) and DAPI (VECTESHIELD Mounting Medium with DAPI, Vector Laboratories, Burlingame, CA, USA). Digital images were captured using a Keyence BZ-9000 microscope (Keyence Co., Osaka, Japan).

### IF of testis

Testes were collected and fixed in 4% (w/v) paraformaldehyde/PBS overnight and embedded in paraffin. Sections were probed with the primary antibody (anti-TLR7 antibody, anti-TLR8 antibody, and anti-sp56 antibody [#55101; QED Biologicals, La Jolla, CA] were used at 1:100). After washing by 0.3% (v/v) Triton X-100 in PBS (−), the antigens were visualized with Cy3-conjugated goat anti-rabbit IgG (1:200, Sigma), FITC-conjugated goat anti-mouse IgG (1:200, Sigma), and DAPI (VECTESHIELD Mounting Medium with DAPI, Vector Laboratories, Burlingame, CA, USA). Digital images were captured using a Keyence BZ-9000 microscope (Keyence Co., Osaka, Japan) with 20x objective.

### Percoll density gradient centrifugation

For preparing Leydig cells, sperm cell, and Sertoli cells, Percoll density gradient centrifugation was performed as previously described by Umehara and colleagues [52]. Briefly, 10 decapsulated testes from male mice were incubated for 10 min at 37 °C with gentle stirring in 50 ml DMEM (Nacalai Tesque) supplemented with 0.1% BSA (Sigma) and 0.5 mg/ml collagenase (GIBCO). Centrifugation was performed using a discontinuous density gradient of Percoll (20%, 30%, 50%, and 60%; GE Healthcare UK Ltd., Amersham Place, UK). The gradient was centrifuged at 2,500 × g for 60 min at 4 °C, as low temperature significantly prevents cell aggregation. Following centrifugation, the gradient was fractionated into plastic tubes and each fraction was centrifuged at 200 × g for 10 min at 4 °C, then washed twice with cold DMEM and finally centrifuged again at 200 × g for 10 min at 4 °C. Germ cells at different developmental stages including spermatozoa were fractioned at the interphase between 20% and 30% of Percoll. Sertoli cells were between 30% and 50% of Percoll, and Leydig cells were present through the 50% Percoll zone.

### Flowcytometric analysis of TLR7 and TLR8

Sperm were collected from the cauda of the epididymis with a 27-G syringe in HTF medium. Additionally, the sperm cells localized in seminiferous tubule were collected from testis using 0.1% collagenase contained HTF medium. After incubation for 30 min and centrifuging, the pellet was washed using 5% (w/v) BSA/PBS. The sperm or sperm cells were fixed with 100% methanol for 10 min at room temperature and then permeabilized by 0.1% (v/v) TritonX-100/PBS for 10 min. Sperm were incubated in 5% (w/v) BSA/PBS for 30 and then probed with the primary antibody (anti-TLR7 antibody conjugated Alexa fluor 647 [bs-6601R-A647,Bioss], anti-TLR8 antibody, or anti-acetylated tubulin antibody [#T7451; Sigma] were used at 1:100). After washing by 5% (w/v) BSA/PBS, the antigens were visualized with Alexa fluor 488 anti-rabbit IgG (1:200; # 4030–30, Southern Biotech, Birmingham, AL, USA), Alexa fluor 647 anti-mouse IgG (1:200; #ab150115, Abcam), or DAPI (VECTESHIELD Mounting Medium with DAPI, Vector Laboratories, Burlingame, CA, USA). The intensity was analyzed by flow cytometry (Attune NxT Acoustic Focusing Cytometer, Invitrogen).

### The measurement of ATP concentrations in sperm

After culture, intracellular levels of ATP in sperm were measured with Enzylight ATP Assay Kit (Bioassay System, Hayward, USA; cat #: EATP-100) according to the manufacturer’s protocol. Briefly, the sperm samples were homogenized in 100 μL of cold PBS and then centrifuged at 14,000 x g for 5 min. Each supernatant was transferred to a separate well. Assay buffer and substrate were added at room temperature. After mixing by tapping the plate, the luminescence was read on a luminometer (2030 Multilabel Reader ARVO X4, PerkinElmer Inc, Waltham, MA, USA) within 1 min after adding.

### The measurement of mitochondrial activity in sperm

Sperm mitochondrial activity was measured with MitoPT JC-1 Assay Kit (911, ImmunoChemistry Technologies, llc.) according to Zhu and colleagues (2019) [40]. Briefly, sperm samples were incubated with 500 μL 1x working solution at 37 °C for 30 min in dark, the mitochondrial activity was analyzed by flow cytometry using a filter with a bandwidth of 574/26 nm (Attune NxT Acoustic Focusing Cytometer, Invitrogen) and measured as the mean fluorescence intensity (MFI) of JC-1 orange aggregates. A total of 50,000 sperm events were analyzed.

### The measurement of the uptake of glucose in sperm

The sperm were incubated in HTF medium with/without 0.3 μM R848 for 30 min. After 1 mM 2-deoxyglucose (2DG) was added to the medium, the sperm were further incubated for 30 min. The sperm samples were collected by the centrifuging at 14,000 x g for 5 min. The uptakes of glucose in sperm were measured using 2-deoxyglucose Uptake Measurement Kit (Cosmo Bio, Japan) according to manufacturer’s protocol.

### Statistics

Statistical analyses of data from three or four replicates for comparison were carried out by either Student *t* test or one-way ANOVA followed by Student *t* test (Statview; Abacus Concepts, Inc., Berkeley, CA).

## Supporting information

S1 Text Supporting information of Materials and methods. (DOCX)

S1 Table The fertilization, development, and XX/XY rates in mouse embryo after IVF using TLR7/8 ligands, R848. IVF, in vitro fertilization; TLR7/8, Toll-like receptor 7/Toll-like receptor 8; R848, Resiquimod.
(DOC)

S2 Table List of primers employed for sexing of mouse embryo. (DOC)

S3 Table List of primers employed for RT-PCR and the expected size. RT-PCR, reverse transcription PCR.
(DOC)

S1 Fig Expression of the genes encoding the X-linked receptors in mouse sperm. Sperm were collected from the cauda epididymis, and were incubated for 30 min in HTF medium. After that, the upper-sperm were collected to remove the other cells. RNA from the sperm was extracted for RT-PCR analyses using specific primer sets. RNA prepared from spleen was used as PC of *Tlr8* and *Tlr7*, and testis was used as positive control of *Ar*. RNA prepared from brain was used as positive control of *Gpr174*, *Gpr34*, and *Edr2a*. RNA prepared from epidydimis was used as positive control of *Hoxa1* and *Hoxb1*. cDNA products were resolved on 2% (w/v) agarose gels. cDNA, complementary DNA; HTF, human tubal fluid; PC, positive control; RT-PCR, reverse transcription PCR; TLR7/8, Toll-like receptor 7/Toll-like receptor 8.
(TIF)

S2 Fig The expression of TLR7 in of mouse sperm cells collected from seminiferous tubule. (A) Density plot between TLR7 and sp56 of mouse sperm cells collected from seminiferous tubule. Sperm cell were collected using percoll method, and incubated with the anti-TLR7 antibody and anti-sp56 antibody, that is a marker of round spermatid, and then were used for flowcytometric analysis. (B) Percent of TLR7-positive and sp56 positive sperm (TLR7^+^/sp56^+^) and TLR7-negative and sp56-positive sperm (TLR7−/sp56^+^). The experiment was repeated three times using a total of three male mice. Values represent the mean ± SEM of three replicates. Data associated with this figure can be found in the supplemental data file (S1 Data). TLR7, Toll-like receptor 7.
(TIF)

S3 Fig Localization of TLR7 and X chromosome in sperm cell. Sperm cells were collected from seminiferous tubule by percoll method, and were mounted on glass slides and air-dried. After incubation in 10 mM citric acid (pH 10.0), the slides were incubated with the probe of X chromosome (X-paint, Creative bioarrays) for 24 hrs. The slides were washed and probed with anti-TLR7 antibody, and the antigens were visualized with Cy3-conjugated goat anti-rabbit IgG. Digital images were captured using a Keyence BZ-9000 microscope. Scale bar indicated 10 μm. White arrows indicated TLR7-negative and X-paint–negative cells. Yellow arrows indicated TLR7-positive and X-paint–positive cells. Green arrows indicated TLR7-negative and X-paint–positive cells. Cy3, Cyanine 3; IgG, Immunoglobulin G; TLR7, Toll-like receptor 7.
(TIF)

S4 Fig Negative control for whole-mount IF of mouse sperm. Sperm were collected from the epididymis into HTF medium and then air dried. Smears were incubated Cy3-tagged anti-rabbit IgG goat antibody. Scale bar indicated 10 μm. Cy3, Cyanine 3; HTF, human tubal fluid; IF, immunofluorescence; IgG, Immunoglobulin G.
(TIF)

S5 Fig The method of IVF using the sperm separated by the treatment with TLR7/8 ligand. Sperm collected from the epididymis were incubated in 1 mL HTF medium with/without 0.3 μM R848 for 60 min. After incubation, the upper-layer was transferred to a new tube and centrifuged at 37 °C for 5 min. The pellet was suspended in HTF medium without R848, and re-centrifuged. After removing the supernatant, the pellet was suspended and transferred to fertilization medium at final number of 1,000 spermatozoa per COC. COC, cumulus-oocyte complex; HTF, human tubal fluid; IVF, in vitro fertilization; TLR7/8, Toll-like receptor 7/Toll-like receptor 8.
(TIF)

S6 Fig The effects of R848 on the sperm apoptosis and the acrosome status. (A) Percentage of TUNEL-positive sperm after incubation with R848. Sperm collected from the epididymis were incubated with 0.3 μM R848 for 60 min. Values are the mean ± SEM of three replicates. *P < 0.05 compared with the control. Data associated with this figure can be found in the supplemental data file (S1 Data). (B) Percentage of acrosome-intact sperm after incubation with R848. Sperm collected from the epididymis were incubated with 0.3 μM R848 for 60 min. Sperm were air dried and then incubated with PNA-FITC in PBS, a known marker of intact sperm acrosomes. Values are the mean ± SEM of three replicates. Data associated with this figure can be found in the supplemental data file (S1 Data). PNA-FITC, peanut agglutinin lectin; R848, Resiquimod; TUNEL, TdT-mediated dUTP Nick End Labeling.
(TIF)

S7 Fig The recovery of sperm motility suppressed by R848 after washing by centrifugation. (A) Tracks of lower-layer sperm incubated with 0.3 μM R848 for 1 hr and lower-layer sperm after washing using ligand-free medium. White arrows indicated the progressive sperm (VAP > 70 mm/sec). Yellow arrows indicated the slow sperm (VAP < 30 mm/sec). (B) The effect of washing using ligand-free medium on VAP of sperm after R848 treatment. Sperm were incubated with 0.3 μM R848 for 1 hr, and then lower-layer sperm were collected to a new tube. After centrifuging, the pellet was washed ligand-free medium twice. Using CASA system, the VAP of sperm before/after washing was compared. The experiment was repeated four times. Values represent the mean ± SEM. *Denotes significant differences between treatments. Data associated with this figure can be found in the supplemental data file (S1 Data). CASA, computer-assisted sperm analysis; R848, Resiquimod; VAP, average-path velocity.
(TIF)

S8 Fig The rates of fertilization and the development to the blastocyst stage when sperm were treated with R848. (A,B) Fertilization (A) and embryo development to the blastocyst stage (B) using sperm treated with R848. About 20 ovulated COCs were placed in 50 μL HTF medium. Sperm were collected and treated according to S5 Fig, and then were transferred to fertilization medium with oocytes. At 6 hrs after insemination, some oocytes were examined for the number of PN. Other oocytes were cultured further in the developing medium to assess development to the blastocyst stage. Values are the mean ± SEM of five replicates. *P < 0.05 compared with the control. (C) Litter size delivered from embryo transfer. Thirty blastocysts at 3.5 days after insemination were surgically transferred into the uterine horns of 2.5-day-old pseudo-pregnant females. The number of pups was then recorded at birth. Values are the mean ± SEM of three replicates. COC, cumulus-oocyte complex; HTF, human tubal fluid; PN, pro-nuclei.
(TIF)

S1 Data All individual numerical values that underlie the summary data that are shown in figures. (XLSX)

## Abbreviations

CASA: computer-assisted sperm analysis
COC: cumulus-oocyte complex
FCM: flow cytometry
HBV: hepatitis B virus
HTF: human tubal fluid
IF: immunofluorescence
IVF: in vitro fertilization
MFI: mean fluorescence intensity
PN: pro-nuclei
RT-PCR: reverse transcription-polymerase chain reaction
TCA: tricarboxylic acid
TLR7/8: Toll-like receptor 7/Toll-like receptor 8
VAP: average-path velocity
X-sperm: X chromosome–bearing sperm
Y-sperm: Y chromosome–bearing sperm
