# Supplementary material for: Activation of Toll-like receptor 7/8 encoded by the X chromosome alters sperm motility and provides a novel simple technology for sexing sperm
Source: PLoS Biol. 2019 Aug 13;17(8):e3000398. doi: 10.1371/journal.pbio.3000398 (PMC6691984; doi:10.1371/journal.pbio.3000398)
Supplement: S1 Text — (DOCX) [file pbio.3000398.s001.docx]

Peanut agglutinin lectin (PNA) staining of sperm

To detect the presence of intact acrosomes, sperm were mounted on glass slides and air-dried. The sperm were permeabilized by 0.1% (v/v) TritonX-100/PBS and fixed with 100% methanol for 10 min at room temperature. After washing in PBS, sperm were incubated with PNA-FITC (Sigma-Aldrich) in PBS for 10 min at 37 °C and washed three times for 5 min in PBS. The fluorescent images were captured using Keyence BZ-9000 microscope (Keyence Co, Osaka, Japan).

TUNEL assay of sperm

Analyses of apoptotic cells (containing fragmented DNA) were performed by the terminal deoxynucleotidyl transferase (Tdt)-mediated biotinylated deoxyuridine triphosphates (dUTP) nick end-labeling (TUNEL) method (In Situ Cell Death Detection Kit POD, Roche Diagnostics) according to the manufacturer's instructions. Sperm were air-dried on the slides and the sperm were permeabilized by 0.1% (v/v) TritonX-100/PBS and fixed with 100% methanol for 10 min at room temperature. After washing with PBS, the positive control sample (but not experimental samples) was treated with DNase (5 μg/mL) for 15 min at 4 °C. Then, all tissue samples were rinsed in PBS and incubated with TUNEL reaction mixture at 37 °C for 60 min. The fluorescent images were captured using Keyence BZ-9000 microscope (Keyence Co, Osaka, Japan).

Immuno-FISH

Sperm cells were collected from seminiferous tubule using Percoll density gradient centrifugation. Sperm cells were mounted on glass slides and air-dried. The slides were heated at 70 °C for 2 hours and then incubated in 10 mM citric acid buffer (pH 10.0) at 90 °C for 30 min. After that, the slides were incubated in 70% formamide/2×Saline Sodium Citrate (SSC) at 70 °C for 2 min. The slides were placed in 70% ethanol at 4 °C for 5 min, transferred to 100% ethanol, placed at room temperature for 5 min, and then air-dried. After the X-chromosomal probe (Mouse Chromosome X Paint Probe, #FMWCP-20, Creative Bioarray, Shirley, NY, USA ) were heated at 75°C for 10 min, and iced on the crash ice for 10 min, the probe mounted on the sample-slide and then incubated at 37 °C for 24 hours. The slides were washed using 2×SSC, and probed with the primary antibody (Anti-TLR7 antibody, Bioss) at 37°C for 2 hour. After washing, the antigens were visualized with Cy3-conjugated goat anti-rabbit IgG (1:200, Sigma) and DAPI (VECTESHIELD Mounting Medium with DAPI, Vector Laboratories). Digital images were captured using a Keyence BZ-9000 microscope (Keyence Co).
