# Supplementary material for: Activation of Toll-like receptor 7/8 encoded by the X chromosome alters sperm motility and provides a novel simple technology for sexing sperm
Source: PLoS Biol. 2019 Aug 13;17(8):e3000398. doi: 10.1371/journal.pbio.3000398 (PMC6691984; doi:10.1371/journal.pbio.3000398)
Supplement: S1 Table — IVF, in vitro fertilization; TLR7/8, Toll-like receptor 7/Toll-like receptor 8; R848, Resiquimod. (DOC) [file pbio.3000398.s002.doc]

|  |  |  |  |  |  | XX/XY ratio | | | |
| --- | --- | --- | --- | --- | --- | --- | --- | --- | --- |
|  | Total oocyte | 2-cell | (%) | Blastocysts | (%) | XX | (%) | XY | (%) |
| Control | 127 | 86 | (70%) | 56 | (64%) | 29 | (53%) | 27 | (47%) |
| Up | 158 | 113 | (72%) | 77 | (68%) | 9 | (10%) | 68 | (90%) |
| Low | 170 | 122 | (72%) | 83 | (68%) | 58 | (70%) | 25 | (30%) |
