# Supplementary material for: Activation of Toll-like receptor 7/8 encoded by the X chromosome alters sperm motility and provides a novel simple technology for sexing sperm
Source: PLoS Biol. 2019 Aug 13;17(8):e3000398. doi: 10.1371/journal.pbio.3000398 (PMC6691984; doi:10.1371/journal.pbio.3000398)
Supplement: S2 Table — (DOC) [file pbio.3000398.s003.doc]

| Gene | Forward Primer | Reverse Primer | Size | Annealing  temperature |
| --- | --- | --- | --- | --- |
| *Ms-X* | 5’- CAGAGGAAGAGGAAGGCACG-3’ | 5’- CCTGCTCATAGTAGTGGCCG-3’ | 143 | 61 |
| *Ms-Y* | 5’- CGGTGTTTGGCGTGAAATGT-3’ | 5’- AACTGTTGTCCGTAGAGCCG-3’ | 178 | 61 |
