# Supplementary material for: Activation of Toll-like receptor 7/8 encoded by the X chromosome alters sperm motility and provides a novel simple technology for sexing sperm
Source: PLoS Biol. 2019 Aug 13;17(8):e3000398. doi: 10.1371/journal.pbio.3000398 (PMC6691984; doi:10.1371/journal.pbio.3000398)
Supplement: S3 Table — RT-PCR, reverse transcription PCR. (DOC) [file pbio.3000398.s004.doc]

| Gene | Forward Primer | Reverse Primer | Size | Annealing  temperature |
| --- | --- | --- | --- | --- |
| *Tlr8* | 5’-GAAGCATTTCGAGCATCTCC-3’ | 5’-GAAGACGATTTCGCCAAGAG-3’ | 188 | 60 |
| *Ar* | 5’-TGAAATGGGACCTTGGATGG-3’ | 5’-GTTTCCCTTGCGCAGCTCTT-3’ | 209 | 60 |
| *Gpr174* | 5’-GCTCACCCACTTGAAAACGG-3’ | 5’-AGCCACTCCAATGAGGATGC-3’ | 142 | 60 |
| *Tlr7* | 5’-GGAAATTGCCCTCGATGTTA-3’ | 5’-CAAAAATTTGGCCTCCTCAA-3’ | 237 | 60 |
| *Gpr34* | 5’- CTGCCTCCCTTTCCGCATAA-3’ | 5’-TGCTTGGTGGTTATTGCCCT-3’ | 195 | 60 |
| *Edr2a* | 5’-AGCGATAAGTAGATGTGGCCC-3’ | 5’-TGCACACAGCAAGAGGTGAT-3’ | 121 | 60 |
| *Tlr8-intron spanning* | 5’-TTGCCAAAGTCTGCTCTCTG-3’ | 5’-TGACTGAGGGGGCATGTTTT-3’ | 127 | 60 |
| *Tlr7-intron spanning* | 5’-TCCTCCACCAGACCTCTTGA-3’ | 5’-TGTCTCTTGCTGCCCCAAAC-3’ | 117 | 60 |
| *Hoxa1* | 5’-GCTGGACTACAGTGGTTGCT-3’ | 5’-TCTTCCAGGGAAAGCTGCAA-3’ | 123 | 55 |
| *Hoxb1* | 5’-GACCGCAACCTTTGCATCAG-3’ | 5’-CGGACACCTTCGCTGTCTTA-3’ | 143 | 55 |
