# Supplementary figures and images for: Activation of Toll-like receptor 7/8 encoded by the X chromosome alters sperm motility and provides a novel simple technology for sexing sperm
Source: PLoS Biol. 2019 Aug 13;17(8):e3000398. doi: 10.1371/journal.pbio.3000398 (PMC6691984; doi:10.1371/journal.pbio.3000398)

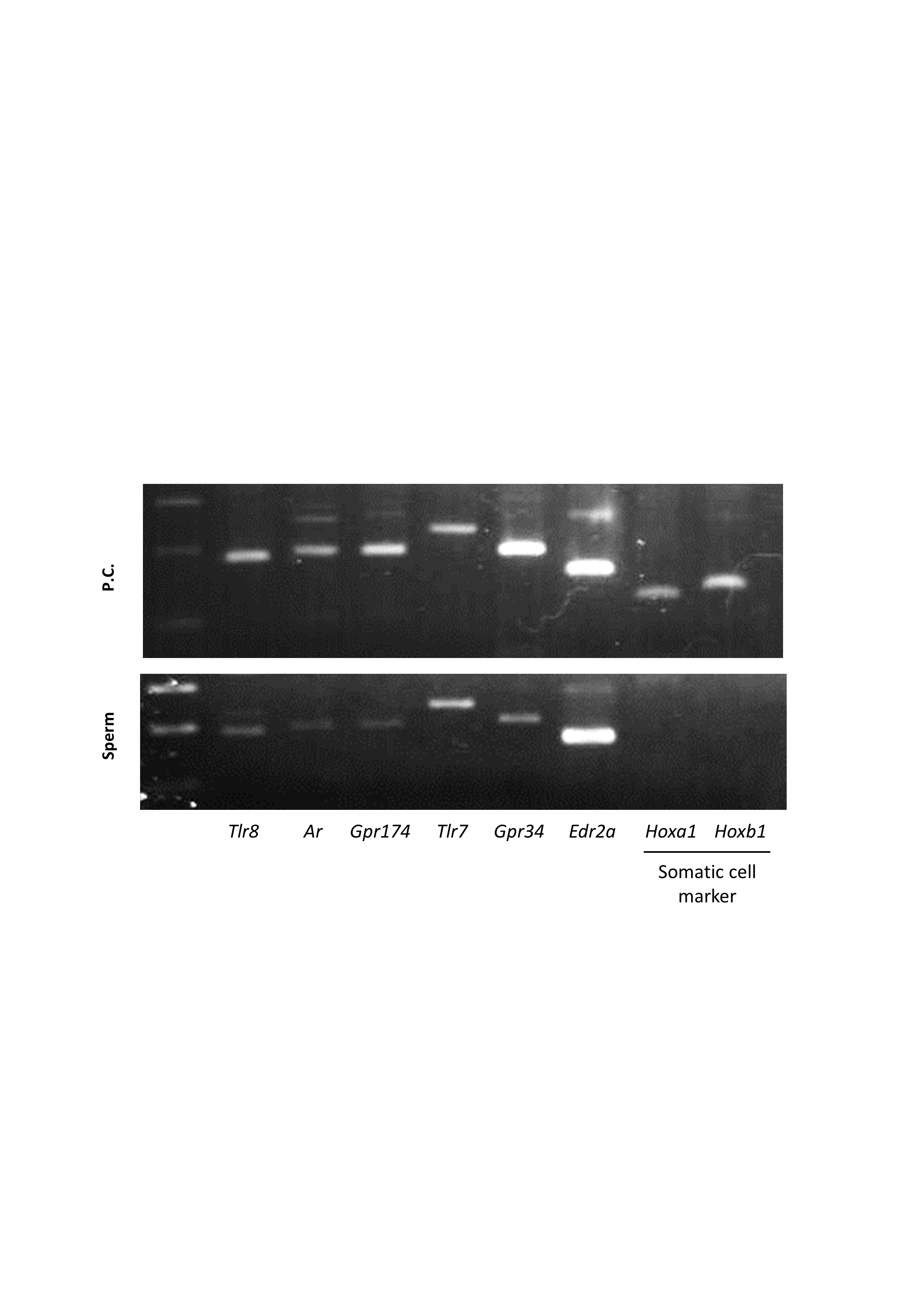

Supplement: S1 Fig — Sperm were collected from the cauda epididymis, and were incubated for 30 min in HTF medium. After that, the upper-sperm were collected to remove the other cells. RNA from the sperm was extracted for RT-PCR analyses using specific primer sets. RNA prepared from spleen was used as PC of Tlr8 and Tlr7, and testis was used as positive control of Ar. RNA prepared from brain was used as positive control of Gpr174, Gpr34, and Edr2a. RNA prepared from epidydimis was used as positive control of Hoxa1 and Hoxb1. cDNA products were resolved on 2% (w/v) agarose gels. cDNA, complementary DNA; HTF, human tubal fluid; PC, positive control; RT-PCR, reverse transcription PCR; TLR7/8, Toll-like receptor 7/Toll-like receptor 8. (TIF) [file pbio.3000398.s005.TIF]

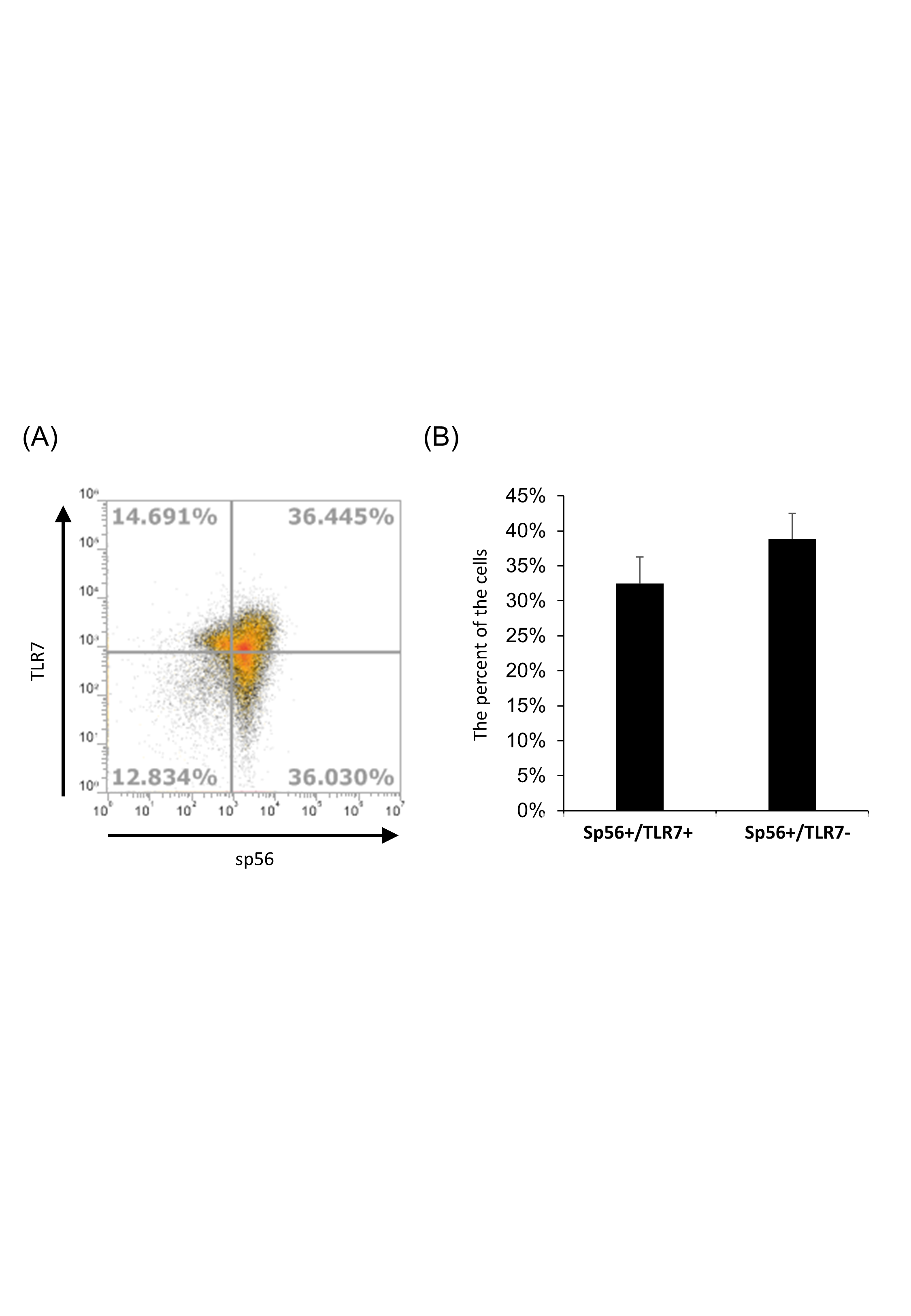

Supplement: S2 Fig — (A) Density plot between TLR7 and sp56 of mouse sperm cells collected from seminiferous tubule. Sperm cell were collected using percoll method, and incubated with the anti-TLR7 antibody and anti-sp56 antibody, that is a marker of round spermatid, and then were used for flowcytometric analysis. (B) Percent of TLR7-positive and sp56 positive sperm (TLR7+/sp56+) and TLR7-negative and sp56-positive sperm (TLR7−/sp56+). The experiment was repeated three times using a total of three male mice. Values represent the mean ± SEM of three replicates. Data associated with this figure can be found in the supplemental data file (S1 Data). TLR7, Toll-like receptor 7. (TIF) [file pbio.3000398.s006.tif]

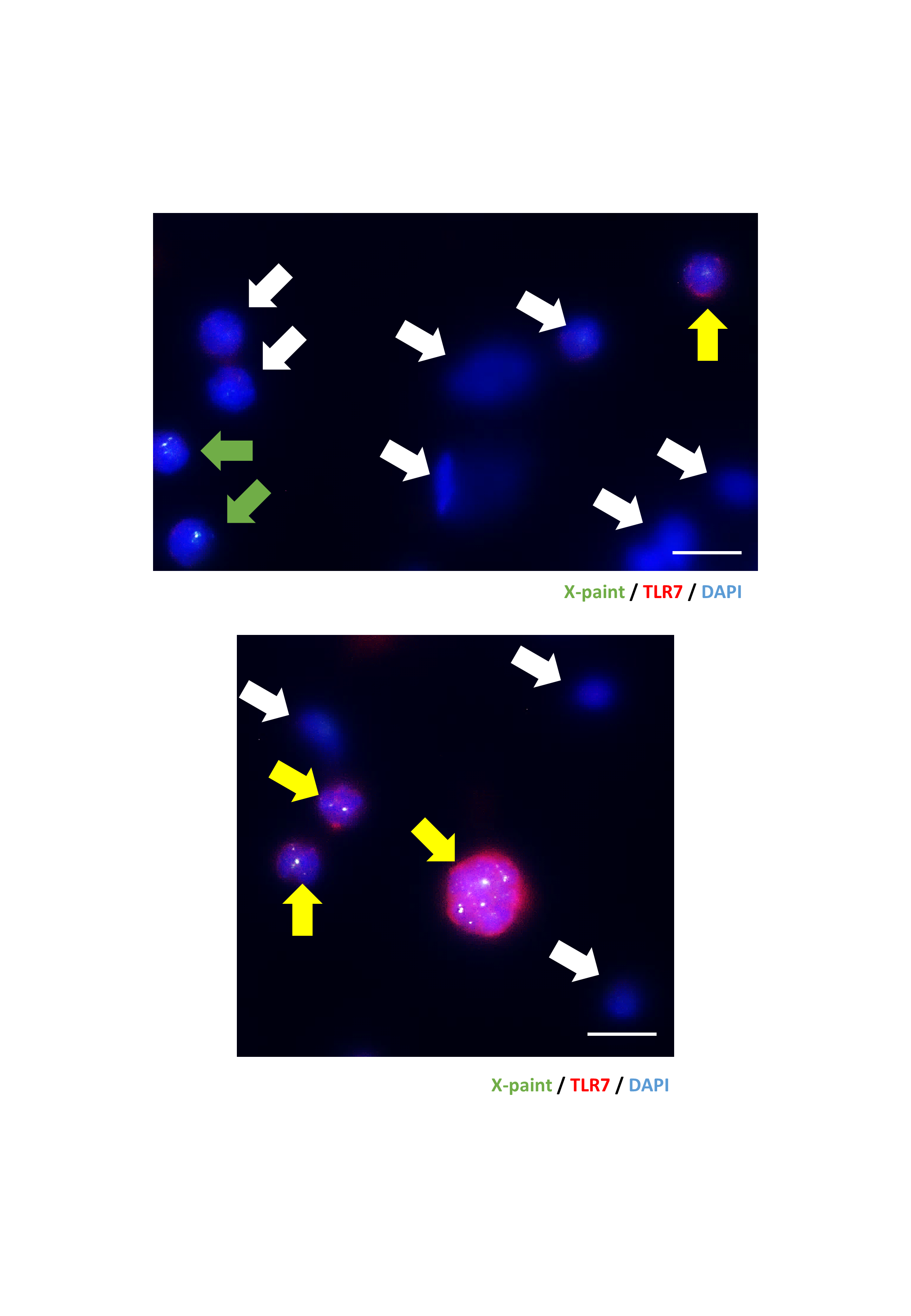

Supplement: S3 Fig — Sperm cells were collected from seminiferous tubule by percoll method, and were mounted on glass slides and air-dried. After incubation in 10 mM citric acid (pH 10.0), the slides were incubated with the probe of X chromosome (X-paint, Creative bioarrays) for 24 hrs. The slides were washed and probed with anti-TLR7 antibody, and the antigens were visualized with Cy3-conjugated goat anti-rabbit IgG. Digital images were captured using a Keyence BZ-9000 microscope. Scale bar indicated 10 μm. White arrows indicated TLR7-negative and X-paint–negative cells. Yellow arrows indicated TLR7-positive and X-paint–positive cells. Green arrows indicated TLR7-negative and X-paint–positive cells. Cy3, Cyanine 3; IgG, Immunoglobulin G; TLR7, Toll-like receptor 7. (TIF) [file pbio.3000398.s007.TIF]

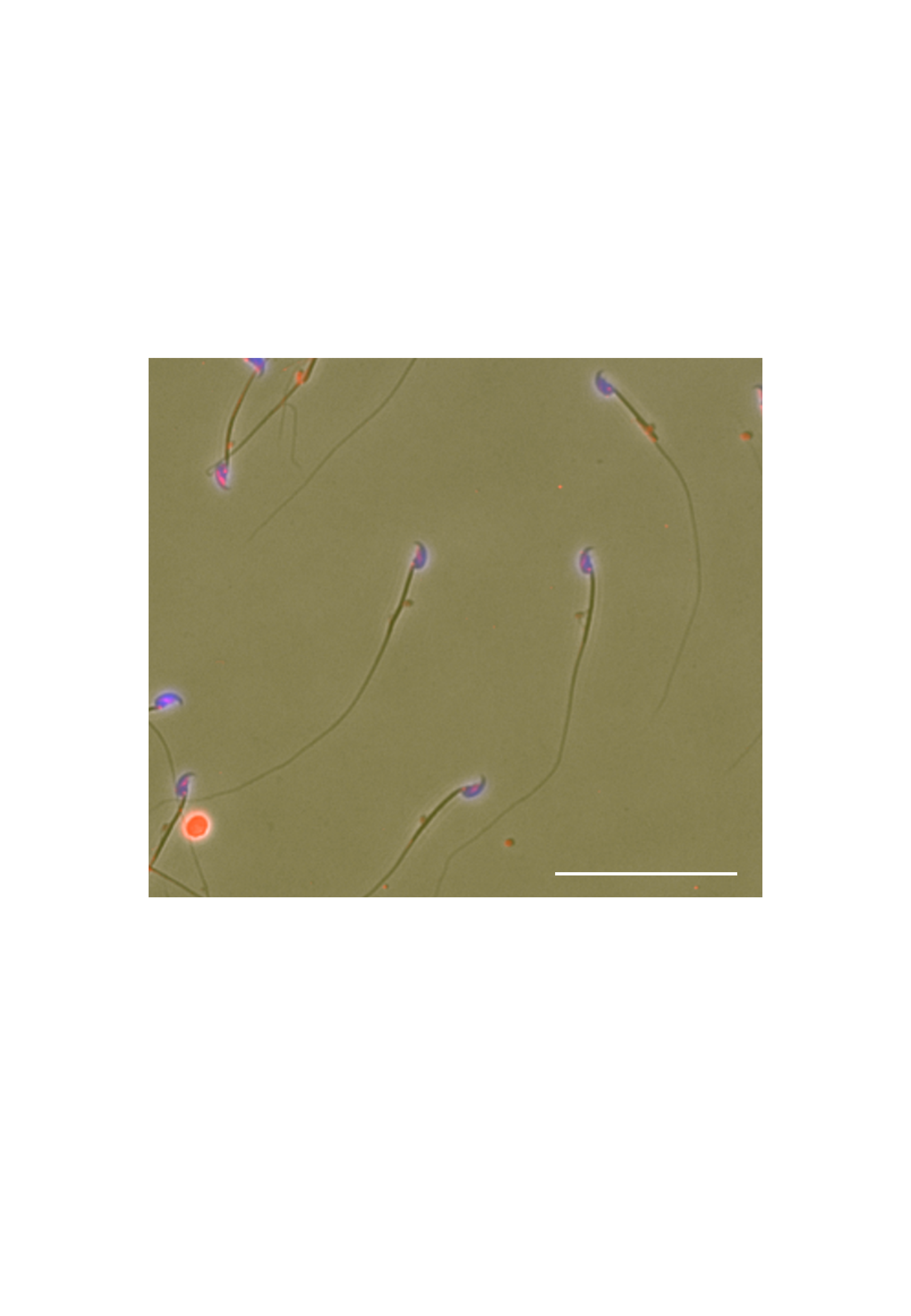

Supplement: S4 Fig — Sperm were collected from the epididymis into HTF medium and then air dried. Smears were incubated Cy3-tagged anti-rabbit IgG goat antibody. Scale bar indicated 10 μm. Cy3, Cyanine 3; HTF, human tubal fluid; IF, immunofluorescence; IgG, Immunoglobulin G. (TIF) [file pbio.3000398.s008.TIF]

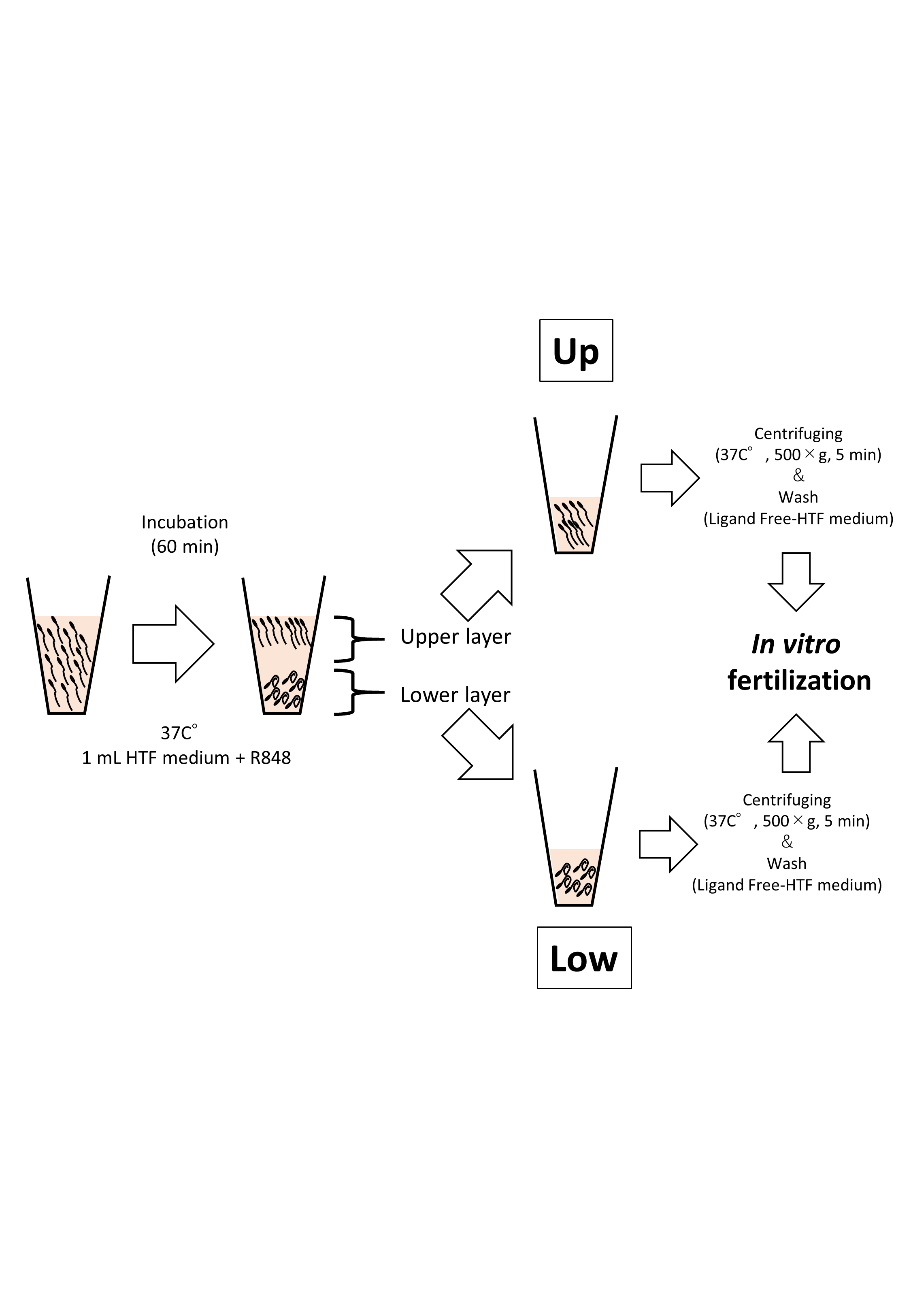

Supplement: S5 Fig — Sperm collected from the epididymis were incubated in 1 mL HTF medium with/without 0.3 μM R848 for 60 min. After incubation, the upper-layer was transferred to a new tube and centrifuged at 37 °C for 5 min. The pellet was suspended in HTF medium without R848, and re-centrifuged. After removing the supernatant, the pellet was suspended and transferred to fertilization medium at final number of 1,000 spermatozoa per COC. COC, cumulus-oocyte complex; HTF, human tubal fluid; IVF, in vitro fertilization; TLR7/8, Toll-like receptor 7/Toll-like receptor 8. (TIF) [file pbio.3000398.s009.TIF]

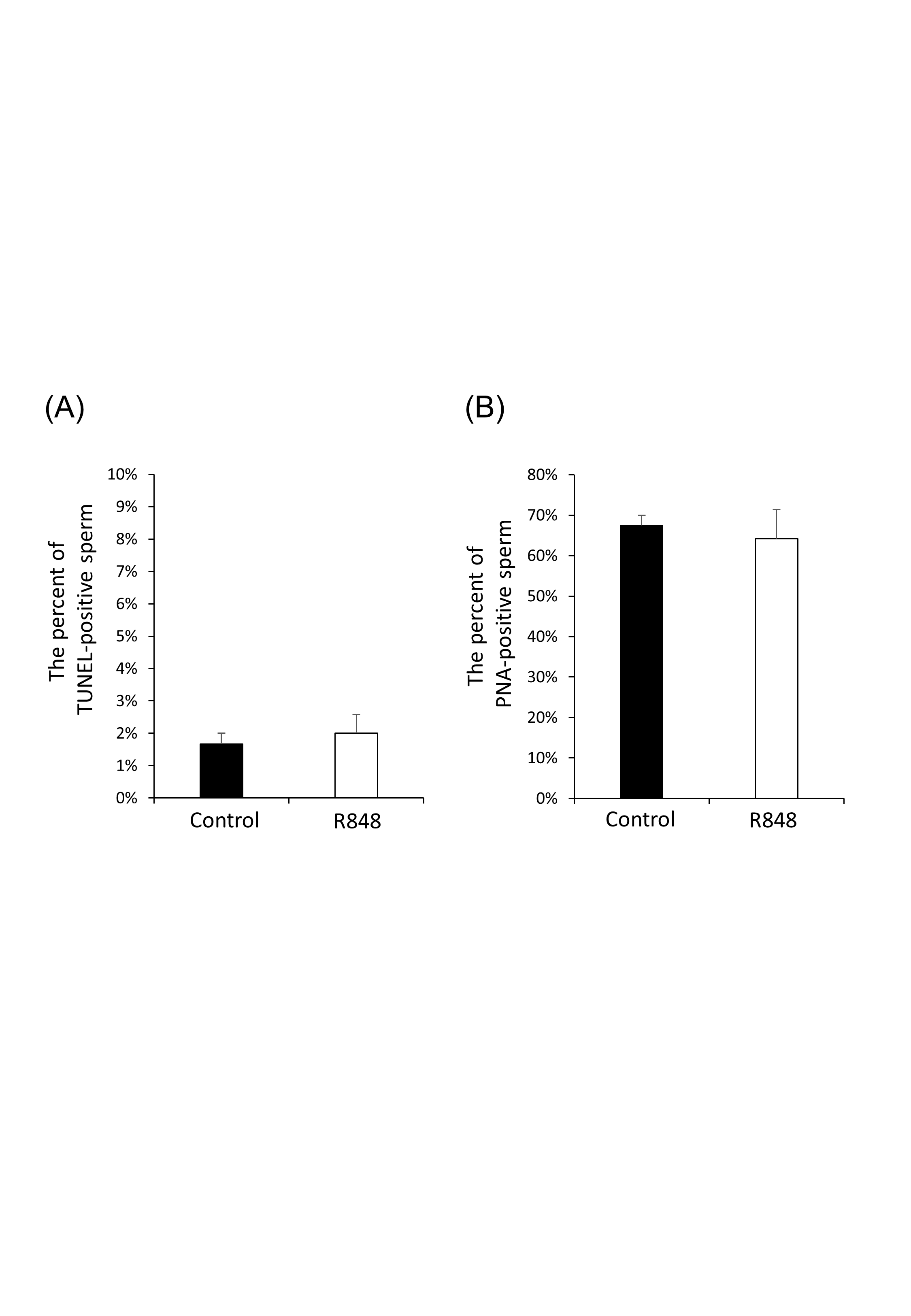

Supplement: S6 Fig — (A) Percentage of TUNEL-positive sperm after incubation with R848. Sperm collected from the epididymis were incubated with 0.3 μM R848 for 60 min. Values are the mean ± SEM of three replicates. *P < 0.05 compared with the control. Data associated with this figure can be found in the supplemental data file (S1 Data). (B) Percentage of acrosome-intact sperm after incubation with R848. Sperm collected from the epididymis were incubated with 0.3 μM R848 for 60 min. Sperm were air dried and then incubated with PNA-FITC in PBS, a known marker of intact sperm acrosomes. Values are the mean ± SEM of three replicates. Data associated with this figure can be found in the supplemental data file (S1 Data). PNA-FITC, peanut agglutinin lectin; R848, Resiquimod; TUNEL, TdT-mediated dUTP Nick End Labeling. (TIF) [file pbio.3000398.s010.TIF]

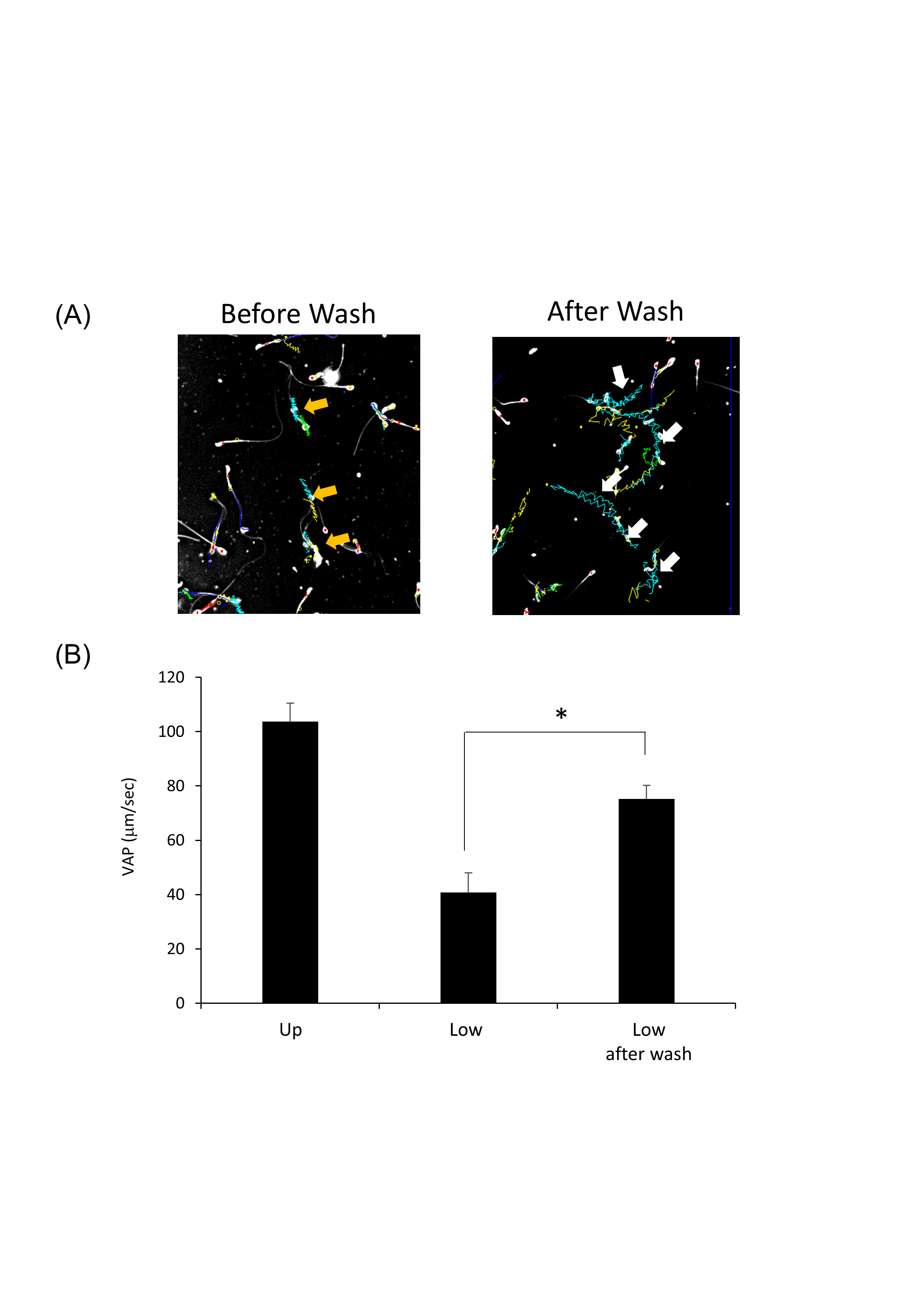

Supplement: S7 Fig — (A) Tracks of lower-layer sperm incubated with 0.3 μM R848 for 1 hr and lower-layer sperm after washing using ligand-free medium. White arrows indicated the progressive sperm (VAP > 70 mm/sec). Yellow arrows indicated the slow sperm (VAP < 30 mm/sec). (B) The effect of washing using ligand-free medium on VAP of sperm after R848 treatment. Sperm were incubated with 0.3 μM R848 for 1 hr, and then lower-layer sperm were collected to a new tube. After centrifuging, the pellet was washed ligand-free medium twice. Using CASA system, the VAP of sperm before/after washing was compared. The experiment was repeated four times. Values represent the mean ± SEM. *Denotes significant differences between treatments. Data associated with this figure can be found in the supplemental data file (S1 Data). CASA, computer-assisted sperm analysis; R848, Resiquimod; VAP, average-path velocity. (TIF) [file pbio.3000398.s011.TIF]

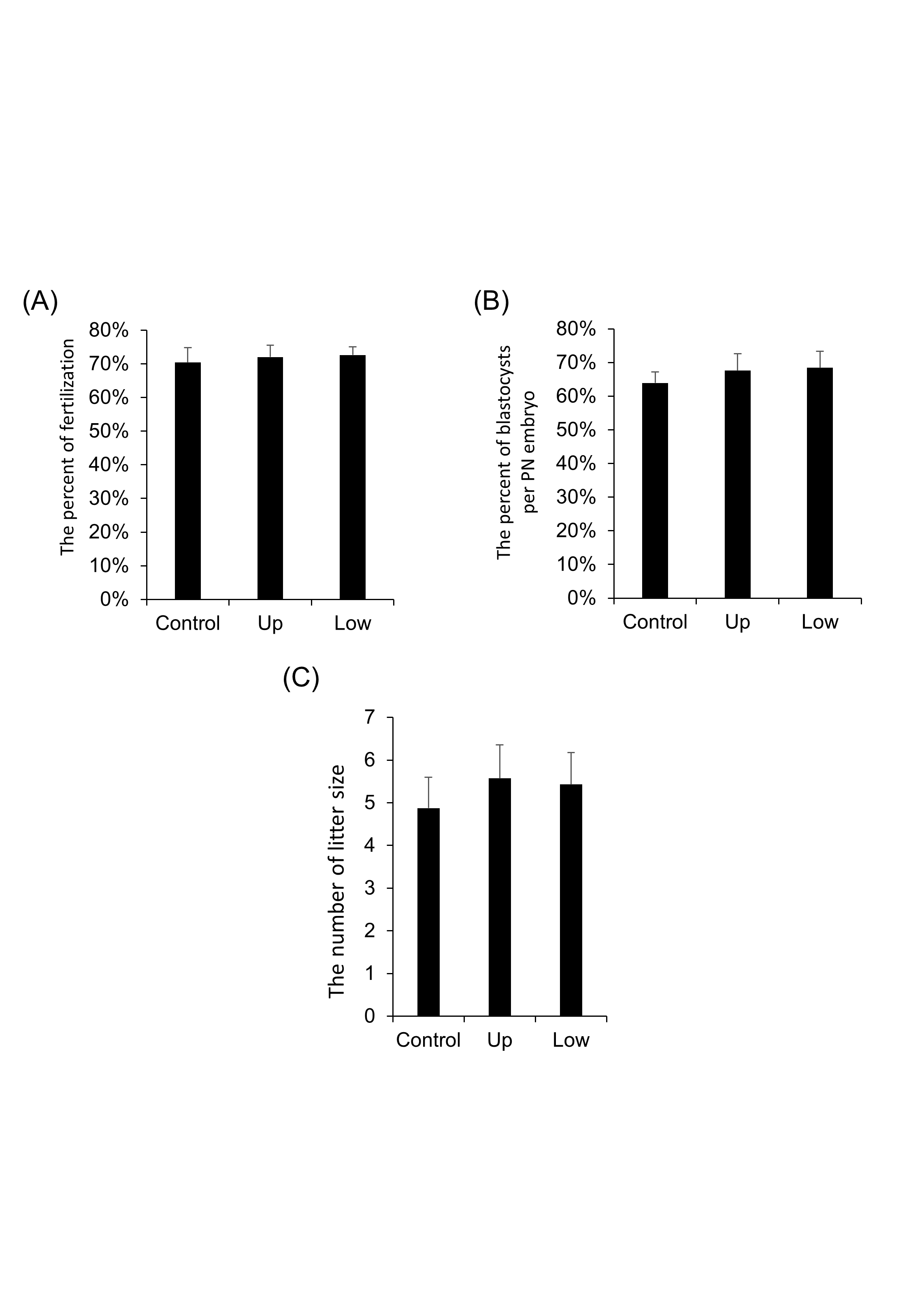

Supplement: S8 Fig — (A,B) Fertilization (A) and embryo development to the blastocyst stage (B) using sperm treated with R848. About 20 ovulated COCs were placed in 50 μL HTF medium. Sperm were collected and treated according to S5 Fig, and then were transferred to fertilization medium with oocytes. At 6 hrs after insemination, some oocytes were examined for the number of PN. Other oocytes were cultured further in the developing medium to assess development to the blastocyst stage. Values are the mean ± SEM of five replicates. *P < 0.05 compared with the control. (C) Litter size delivered from embryo transfer. Thirty blastocysts at 3.5 days after insemination were surgically transferred into the uterine horns of 2.5-day-old pseudo-pregnant females. The number of pups was then recorded at birth. Values are the mean ± SEM of three replicates. COC, cumulus-oocyte complex; HTF, human tubal fluid; PN, pro-nuclei. (TIF) [file pbio.3000398.s012.TIF]
